# Comparison of phytochemical composition of *Ginkgo biloba* extracts using a combination of non-targeted and targeted analytical approaches

**DOI:** 10.1007/s00216-020-02839-7

**Published:** 2020-08-31

**Authors:** Bradley J. Collins, Season P. Kerns, Kristin Aillon, Geoffrey Mueller, Cynthia V. Rider, Eugene F. DeRose, Robert E. London, James M. Harnly, Suramya Waidyanatha

**Affiliations:** 1 Division of the National Toxicology Program, National Institute of Environmental Health Sciences, Research Triangle Park, NC 27709 USA; 2 MRIGlobal, Kansas City, MO 64110 USA; 3 Division of Intramural Research, National Institute of Environmental Health Sciences, Research Triangle Park, NC 27709 USA; 4 U.S. Department of Agriculture, Beltsville Human Nutrition Research Center, Methods and Applications Food Composition Lab, Beltsville, MD 20705 USA

**Keywords:** Natural products, Non-targeted analysis, *Ginkgo biloba*, Phytochemical characterization, Dietary supplements

## Abstract

*Ginkgo biloba* extract (GbE) is a dietary supplement derived from an ethanolic extract of *Ginkgo biloba* leaves. Unfinished bulk GbE is used to make finished products that are sold as dietary supplements. The variable, complex composition of GbE makes it difficult to obtain consistent toxicological assessments of potential risk. The National Toxicology Program (NTP) observed hepatotoxicity in its rodent studies of a commercially available, unfinished GbE product, but the application of these results to the broader GbE supplement market is unclear. Here, we use a combination of non-targeted and targeted chromatographic and spectrophotometric methods to obtain profiles of 24 commercially available finished GbE products and unfinished standardized and unstandardized extracts with and without hydrolysis, then used principal component analysis to group unfinished products according to their similarity to each other and to National Institute of Standards and Technology (NIST) standard reference materials (SRM), and the finished products. Unfinished products were grouped into those that were characteristic and uncharacteristic of standardized GbE. Our work demonstrates that different analytical approaches produced similar classifications of characteristic and uncharacteristic products in unhydrolyzed samples, but the distinctions largely disappeared once the samples were hydrolyzed. Using our approach, the NTP GbE was most similar to two unfinished GbE products classified as characteristic, finished products, and the NIST GbE SRM. We propose that a simple analysis for the presence, absence, or amounts of compounds unique to GbE in unhydrolyzed samples could be sufficient to determine a sample’s authenticity.

Graphical abstract

**Electronic supplementary material:**

The online version of this article (10.1007/s00216-020-02839-7) contains supplementary material, which is available to authorized users.

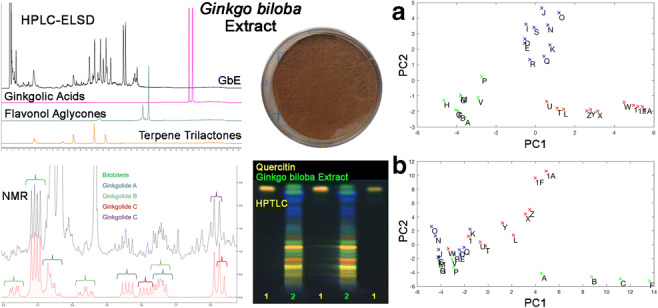

## Introduction

Botanical dietary supplements are complex mixtures of variable composition. The National Toxicology Program (NTP) has evaluated multiple botanical dietary supplements in short-term and long-term toxicity and carcinogenicity studies in rodents [1]. A key challenge identified in the NTP botanical research program is comparing across these complex mixtures, both in the design phase when selecting an appropriate test article from the multitude of available products and subsequently in extending the toxicological evaluation results for a single reference sample to similar products in the marketplace. To address this challenge, the NTP has begun to assess the chemical and biological parameters needed to establish sufficient similarity between complex mixtures, and *Ginkgo biloba* extract (GbE) was selected as the first case study for this assessment [2].

GbE is an ingredient in many commercially available dietary supplements marketed as promoting mental acuity and is sold by a large number of vendors worldwide. GbE is an ethanolic extract of *Ginkgo biloba* leaves with a complex composition. There are two recognized types of GbE: full extracts containing all alcohol-soluble constituents and standardized extracts in which certain constituents are deliberately enriched while others are removed [3]. It is important to note that the multi-step processes involved in preparing standardized GbE from *Ginkgo biloba* leaves are proprietary and can differ from manufacturer to manufacturer [3]. Extracts may be standardized to contain 24% flavonol glycosides consisting primarily of glycosides of quercetin, kaempferol, and isorhamnetin and 6% terpene trilactones, which include ginkgolides A, B, C, and J, and bilobalide, and less than 5 ppm ginkgolic acids [3]. The 24% (flavonol glycosides) to 6% terpene trilactones ratio (abbreviated 24/6) is often listed on finished product labels and certificates of analysis of unfinished products (i.e., the bulk extract that serves as material for finished products often sold as dietary supplements) to indicate that the GbE is standardized [4, 5]. The United States Pharmacopeial Convention specifies an acceptable range of 22.0–27.0% flavonol glycosides and 5.4–12.0% terpene trilactones for Powdered *Ginkgo* Extract [6]. One standardized extract of *Ginkgo biloba* leaves, EGb761®, produced by Dr. Willmar Schwabe GmbH & Co. KG (Karlsruhe, Germany) has become the de facto industry standard and contains 24% of the flavonol glycosides of quercetin, kaempferol, and isorhamnetin and 6% terpene trilactones, including ginkgolides A, B, C, and J and bilobalide. However, surveys of GbE products in the marketplace have found widely variable constituent concentrations [4, 5, 7, 8]. Kressmann et al. surveyed 27 lots of GbE commercially available in the USA and found flavonol glycoside content ranging from 23.88 to 34.54%, terpene trilactone content from 3.87 to 11.31%, and ginkgolic acid content from < 500 to > 89,500 ppm. Typically, flavonol glycoside content is determined after hydrolysis to convert the glycosides to the corresponding aglycones (flavonols); hence, GbE products are often adulterated with cheaper botanical material containing individual flavonol glycosides (e.g., rutin, the glycoside of quercetin) or flavonols (e.g., quercetin, the aglycone of rutin) [9].

The emphasis on flavonol glycosides, terpene trilactones, and ginkgolic acids in standardization of GbE is driven by the purported biological activity of these constituent classes, with flavonol glycosides and terpene trilactones associated with pharmacological activity and ginkgolic acids with toxicological activity. The flavonol glycosides have antioxidant activity [10], while the terpene trilactones display platelet-activating receptor antagonism [11], glycine receptor antagonism [12, 13], and γ aminobutyric acid (GABA_A_) receptor antagonism [14]. Ginkgolic acids have been linked to allergenic [15], cytotoxic [16], and mutagenic activity [17].

Due to the potential for widespread human exposure, the NTP conducted 3-month and 2-year toxicity and carcinogenicity studies in mice and rats with an unfinished GbE product and found that the major toxicity targets were the liver, nose, and thyroid gland [18, 19]. The specific GbE unfinished product was selected as the test article based on comparison with an EGb761®-containing product (unpublished). The GbE used in the NTP studies had levels of flavonol glycosides (31.2%), terpene trilactones (15.4%), and ginkgolic acids (10.5 ppm) that exceeded standardized GbE specifications [18].

The focus of this work was to evaluate multiple finished and unfinished GbE products on the market using a variety of analytical and chemometric techniques to assess similarities and differences between products and then use those results to evaluate the unfinished GbE product used in NTP studies. The GbE materials used include the NTP GbE test article, other unfinished GbE products, known adulterants of GbE (e.g., *Sophora japonica*), GbE standard reference materials (SRM), and finished products. The approach described here provides the basis for assessing the chemical similarity of related products, a critical step in understanding toxicological similarity of complex mixtures [2].

## Materials

A single lot (020703) of unfinished GbE was obtained from Shanghai Xing Ling Sci & Tech. Pharmaceutical Co., Ltd. (Shanghai, China) in 2003 for use in NTP toxicity and carcinogenicity studies. Upon receipt, an aliquot was removed to − 20 °C storage (later designated GbE 1F to distinguish it from the remainder of the lot) and the bulk lot was stored at ambient temperature and used in NTP toxicity and carcinogenicity studies in rats and mice (NTP, 2013). In 2014, an additional aliquot from the bulk lot was moved to − 20 °C storage and designated GbE 1. In 2015, the remainder of the bulk lot was moved to − 20 °C storage and designated GbE 1A. To further characterize the NTP samples (1, 1A, and 1F) and determine their relationship to other GbE products, we obtained 20 unfinished GbE products, 4 finished products containing standardized GbE (e.g., EGb761®), and 2 National Institute of Standards and Technology (NIST) SRMs (see Electronic Supplementary Material (ESM) Table S1). Unfinished products were sourced from 15 suppliers in the US market, including standardized and unstandardized materials. An extract meeting the standard will have 24% w/w flavonol glycosides, 6% w/w terpene trilactones, and < 5 ppm ginkgolic acid and is often labeled 24/6/5. Of the 20 lots (A–T) of unfinished products, 10 were standardized on one or more of these compound categories (A, D–F, I, L, N, O, R, and T, but only T was purported to conform to the 24/6/5 standard). Of the other 10 unfinished products, 4 (B, C, H, and M) were described by their extraction ratio, 10:1 or 4:1. The remaining 6 lots were described as *Ginkgo biloba* powder extract (P), *Ginkgo* dry extract (K), *Ginkgo biloba* extract USP (J, S), *Ginkgo* extract (Q), and *Ginkgo biloba* leaf powder (G). The 4 finished products (W–Z) were obtained from the over-the-counter (OTC) marketplace and contained 60 or 120 mg of EGb761®, with W additionally containing 340 mg of Gotu kola. SRMs representative of an unfinished GbE (SRM 3247, U) and a tablet (SRM 3248, V) were obtained from NIST (Gaithersburg, MD). All of the GbE products and SRMs were stored at − 20 °C upon receipt except as noted above for NTP GbE (1, 1A, 1F).

Ginkgolide A, ginkgolide B, ginkgolide C, ginkgolide J, rutin trihydrate, rutin hydrate, quercetin, kaempferol, isorhamnetin, ginkgolic acid C15:1 (GA-I), and ginkgolic acid C17:1 (GA-II) were obtained from Sigma-Aldrich (St. Louis, MO). Kaempferol was obtained from TCI America (Portland, OR). (-)-Bilobalide was obtained from ChromaDex (Irvine, CA). Ginkgotoxin was obtained from Phytolab (Vestenbergsgreuth, Germany) and Cerilliant Corporation (Round Rock, TX). Prepared mixtures of *Ginkgo biloba* terpene trilactones and flavonoids were also obtained from Sigma-Aldrich (St. Louis, MO). The chemical shift standard used in NMR analyses, 4,4-dimethyl-4-silapentane-1-sulfonic acid (DSS), was obtained from Sigma-Aldrich (St. Louis, MO). Other materials and reagents were purchased from commercial sources.

## Methods

We employed a tiered approach to characterizing both the finished and unfinished GbE samples and SRMs. Starting with a non-targeted chromatographic approach using high-performance liquid chromatography (HPLC) with a non-specific, evaporative light scattering detector (ELSD), all samples and SRMs were analyzed to establish their chromatographic profiles with and without hydrolysis. Unhydrolyzed, unfinished product samples that showed responses in retention time ranges corresponding to known GbE constituents were then analyzed using high-performance thin-layer chromatography (HPTLC) against standard reference materials and authentic GbE component standards and known adulterants. HPLC employing different detectors optimized for each GbE component class was then used to perform a targeted analysis and quantitation of known GbE constituents for all finished and unfinished product samples and SRMs. Finally, chemometric analysis of the raw output from the non-targeted analyses was performed to compare finished and unfinished products, including the NTP test article, with each other and with the SRMs.

### Non-targeted analysis using high-performance liquid chromatography (HPLC)-ELSD

Analyses were performed on each GbE product described above without or with acid hydrolysis to convert flavonol glycosides to corresponding aglycones. Samples were prepared singly at 60 mg GbE/mL in either 80:20 ethanol:water (unhydrolyzed) or 64:26:10 ethanol:water:12N HCl (hydrolyzed). Corresponding blanks were prepared without GbE. For commercial products, the GbE concentration on the label was used to determine the starting product weight to achieve the final concentration of 60 mg GbE/mL. Samples to be hydrolyzed were placed in a 90 °C oven for 1 h and then diluted in methanol to a final concentration of 30 mg GbE/mL. All samples and blanks were filtered through a 0.45-μm PTFE syringe.

Samples were analyzed on a Shimadzu (Columbia, MD) LC-2010C HT HPLC, using a Chromolith®, EMD Millipore (Billerica, MA) Performance RP-18e column (100 × 4.6 mm, 2 μm (macropore), 130 Å (mesopore)). The detector was an Alltech 3300 ELSD, BUCHI (New Castle, DE), with an N_2_ gas flow of 1.5 mL/min and a drift tube temperature of 55 °C. The autosampler was maintained at 15 °C; the column was at 35 °C. Mobile phases isopropyl alcohol (A), tetrahydrofuran (B), and 0.1% formic acid in water (C) were used at a flow rate of 1 mL/min. A linear gradient of A:B:C was used from 5:0:95 to 0:13:87 in 15 min, then to 0:40:60 in 35 min, followed by to 0:75:25 in 20 min, hold for 5 min. Total run time was 75 min.

Commercially available constituent standard mixtures of terpene trilactones and flavonoids (100 μg constituent/mL in methanol), along with individually prepared GA-I and GA-II standards (100 μg/mL in methanol), were run with each sample set to establish approximate retention time ranges for each compound class.

### High-performance thin-layer chromatography (HPTLC)

A subset of 17 unfinished product samples (D, E, G, I–L, N–T), NIST SRM (U), finished product (W), and NTP unfinished product (1A) were analyzed by Alkemist Labs (Costa Mesa, CA) to assess their authenticity using HPTLC to compare samples with known GbE standards and adulterants. When multiple extract lots were available from the same vendor, a single lot was selected. In one case, an extract and leaf powder (unfinished products G and I) were available from one vendor so both materials were analyzed. Lots for which initial non-targeted chromatography screens had suggested that no GbE constituents were present were excluded from this analysis. Each sample was prepared by adding 3 mL of 70% ethanol to a 0.3-g aliquot while sonicating, then heating to 70 °C for 30 min. Samples were run on silica gel 60, F_254_, HPTLC plates using two systems. The system 1 mobile phase consisted of ethyl acetate:acetic acid:formic acid:water (10:1.1:1.1:2.6). Samples were run with a GbE SRM (NIST 3247), caffeic acid, rutin, hyperoside, chlorogenic acid, and genistein standards, and *Sophora japonica* (flower), *S. japonica* (fruit), and the testing laboratory’s *G. biloba* leaf samples, along with a methanol solvent blank. System 2 consisted of toluene:ethyl acetate:formic acid (7:3:1). Samples were run against a GbE SRM (NIST 3247), and genistein, isorhamnetin, and quercetin standards and *S. japonica* (flower), *S. japonica* (fruit), and the testing laboratory’s *G. biloba* leaf samples, along with a methanol blank. All samples were run in a CAMAG Automatic Developing Chamber 2 (CAMAG Scientific, Inc., Wilmington, NC) at 35–40% humidity and were visualized at 365 nm with and without natural product reagent (NPR) + polyethylene glycol (PEG).

### Quantitation of marker constituents by nuclear magnetic resonance spectrophotometry (NMR)

A targeted analysis of marker constituent concentrations was performed for all finished and unfinished GbE samples and GbE SRMs. Samples were prepared at 10 mg/mL in [U-^2^H]-DMSO containing 200 μM DSS as a chemical shift and concentration reference. Standards for nine GbE constituents (rutin trihydrate; isorhamnetin; kaempferol; quercetin; ginkgolides A, B, C, and J; and bilobalide) were prepared similarly in [U-^2^H]-DMSO containing 200 μM DSS. Constituent standards were used to create standardized NMR spectra referenced to DSS for comparison with samples using the software Chenomx (Edmonton, Alberta). NMR spectra were acquired using an Agilent 800 MHz DD2 spectrometer with a cryogenically cooled probe using a NOESY sequence (filename: tnnoesy) with 100-ms mixing time, 4-s acquisition, and 1-s pre-saturation recovery time.

### Quantitation of marker constituents by HPLC

HPLC employing evaporative light scattering, ultraviolet, fluorescence, or mass spectrophotometric detection was used to perform a targeted analysis of all finished and unfinished GbE samples and SRMs for 12 known GbE constituents: bilobalide; ginkgolide A, B, C, and J; quercetin; kaempferol; isorhamnetin; rutin trihydrate; ginkgotoxin; and ginkgolic acids I and II.

### Terpene trilactones, flavonols, and ginkgotoxin

Caffeine (internal standard) stock was made at 0.7 mg/mL. A stock solution containing nine constituent standards (bilobalide; ginkgolide A, B, C, and J; quercetin; kaempferol; and rutin trihydrate) was prepared at ~ 100 mg/mL in methanol and diluted in methanol to prepare 6 concentrations ranging from ~ 4 to ~ 76 μg/mL for each constituent. Six isorhamnetin standards were prepared in methanol at concentrations from ~ 4 to ~ 74 μg/mL. A ginkgotoxin stock solution prepared at ~ 1 mg/mL in methanol was diluted to prepare six standard solutions over the concentration range of ~ 2 to ~ 2000 ng/mL. All standard solutions contained caffeine at ~ 0.14 mg/mL. Standards were not hydrolyzed prior to analysis.

Triplicate, approximately 100 mg portions of each GbE finished and unfinished sample or SRM were dissolved in 50 mL of diluent (ethanol:water:12N hydrochloric acid 64:26:10) with sonication. A 4-mL aliquot was mixed with 1 mL of caffeine stock and diluted to 10 mL with methanol. An additional 5-mL aliquot of each solution was hydrolyzed in a 90 °C oven for 1 h; after cooling, a 4-mL aliquot of each solution was mixed with 1 mL of caffeine stock solution and diluted to 10 mL with methanol. A portion of each hydrolyzed and unhydrolyzed sample was filtered through a 0.45-μm PTFE syringe filter into 2 vials each. One vial was used for analysis of terpene trilactones and flavonol aglycones and a second vial was used for analysis of ginkgotoxin.

Finished and unfinished samples, SRMs, and constituent standards were analyzed for terpene trilactones and flavonol aglycones on a Shimadzu (Columbia, MD) LC-2010C HT liquid chromatograph, using a Phenomenex (Torrance, CA) Prodigy ODS 3 column (5 μ, 250 × 4.6 mm, 100 Å pore size). The detector for terpene trilactone quantitation was an Altech 3300 ELSD with an N_2_ gas flow of 1.5 mL/min and a drift tube temperature of 55 °C. The detector for flavonol aglycone quantitation was an integrated Shimadzu UV at a wavelength of 267 nm. Mobile phases water:methanol (90:10 (v:v)) with 0.25% formic acid (A) and methanol with 0.25% formic acid (B) were used at a flow rate of 1 mL/min. A linear gradient of A:B was run from 85:15 to 62:38 in 23 min, then to 54:46 in 2 min, hold for 30 min, followed by to 10:90 in 5 min, hold for 10 min. Total run time was 70 min.

Triplicate finished and unfinished samples, SRMs, and constituent standards were analyzed for ginkgotoxin using the same system as above, using a Phenomenex (Torrence, CA) Intersil/InertClone ODS-3 (3 μ, 150 × 4.6 mm, 100 Å pore size) column. A Shimadzu (Columbia, MD) spectrofluorometric detector, RF-20AXS, with an excitation wavelength of 295 nm and an emission wavelength of 395 nm was used. Mobile phases 5 mM aqueous potassium phosphate and 5 mM aqueous sodium hexanesulfonate, pH adjusted to 2.5 with phosphoric acid (A) and acetonitrile (B), were employed with a column flow rate of 1 mL/min. The linear gradient used (A:B) was 96:4, hold 1 min, then to 70:30 in 12 min, hold 7 min. Total run time was 20 min.

Peak area response ratios of analyte to internal standard and a linear regression equation were used to determine constituent concentrations in each finished and unfinished sample, SRM, and constituent standard. The method was qualified for use through preparation and analysis of GbE constituent standard curves on each analysis day. A correlation coefficient (*r*) ≥ 0.99 was required for the standard curve to be used to quantitate samples. Determined concentrations of standard constituents were compared against nominal concentrations; standards that had lower accuracy (measured as percent relative error, %RE ≤ 25%) were not used in the standard curve. Method measurement limits, constituent standard curve ranges, and correlation coefficients for bilobalide; ginkgolides A, B, C, and J; and isorhamnetin, kaempferol, and quercetin are given in ESM Table S2. To check the accuracy of the method, two samples of the NIST unfinished GbE SRM were analyzed at different times and results of the analyses were compared with the NIST-reported values for each constituent in the hydrolyzed SRM (ESM Table S5). The determined concentration, dilution volume, and the initial sample weight were used to estimate the weight percent of each constituent in each GbE sample. To estimate the weight percent of flavonol glycosides, weight percent of corresponding flavonols quercetin, kaempferol, and isorhamnetin was multiplied by 2.504, 2.588, and 2.437, respectively (INA Method 102.00).

### Ginkgolic acids

A mixed stock standard containing GA-I and GA-II was prepared in methanol at 1 μg/mL. Five concentrations covering the range of ~ 0.001 to ~ 0.5 μg/mL were prepared by diluting aliquots of the stock standard in methanol.

Triplicate finished and unfinished samples and SRMs were prepared by weighing ~ 30 mg of each GbE sample and diluting to 10 mLwith diluent (see above). An ~ 5-mL aliquot was hydrolyzed at 90 °C for 1 h, cooled to room temperature, and diluted to 10-mL with methanol. The other 5-mL aliquot was diluted to 10 mL with methanol. A portion of each hydrolyzed and unhydrolyzed sample was filtered through a 0.45-μm PTFE syringe filter for analysis.

Finished and unfinished samples were analyzed on a Shimadzu (Columbia, MD) LC-20 AD HPLC, using a Phenomenex (Torrance, CA) Prodigy ODS (3) (5 μm 250 × 4.6 mm, 100 Å pore size) column. The detector was an ABSciex (Concord, Ontario) Tandem Triple Quadrupole Mass Spectrometer run in negative turbo ionspray mode. Mobile phases water:methanol (900:100 (v:v)) with 0.1% formic acid (A) and methanol with 0.1% formic acid (B) were employed with a column flow rate of 1 mL/min. The linear gradient (A:B) used was as follows: 85:15, hold 5 min, then to 5:95 in 10 min, hold 20 min. Total run time was 35 min. Transitions monitored were 345 → 301 for GA-I and 373 → 329 for GA-II. The method was qualified for use through preparation and analysis of GA-I and GA-II component standard curves on each analysis day. The criteria for method qualification and analyte quantitation were similar to those for other constituents. Method measurement limits, constituent standard curve ranges, and correlation coefficients are given in ESM Table S2.

### Data analysis

The raw HPLC-ELSD chromatograms from the non-targeted analyses of finished and unfinished products and SRMs were downloaded into Excel files (Microsoft, Inc., Billingham, WA) as 2-dimensional files (intensity vs. retention time). Data were compiled to produce a 3-dimensional data set with intensity as a function of sample (*Y* axis) and retention time (*X* axis). Principal component analysis (PCA) was performed using Solo (Eigenvector Research, Wenatchee, WA). The data for unhydrolyzed and hydrolyzed samples were analyzed separately. The chromatographic data were initially analyzed by PCA without any pre-processing. These data yielded patterns that were difficult to interpret (data not shown). Hence, the following pre-processing of raw chromatogram data was used prior to PCA. The first 4.33 s (1300 points) was excluded to eliminate solvent peaks and the last 5 min of each chromatogram was dropped due to absence of peaks. The first derivative (of a cubic equation fit to 51 data points) was taken to remove the baseline shifts and the derivatized chromatograms were normalized by setting the sum of squares of the data equal to 1.0. The chromatograms were aligned with respect to retention time using the large peaks at 37.33, 38.47, 46.33, and 49.13 min. An additive shift in time was required for alignment, but a multiplicative lengthening or shortening of each chromatogram was not necessary. The resulting truncated, derivatized, and normalized chromatograms are shown in Figs. S1–S4 (see ESM).

Once aligned, HPLC-ELSD/UV data was analyzed by hierarchical clustering performed using the Ward method. Hydrolyzed and unhydrolyzed sample data were analyzed separately. Percent weight data for each constituent were analyzed using JMP software version 13.0.0 (SAS, Cary, NC). A constellation plot was then used to visualize the data.

The NMR spectra were processed with Chenomx and exported to MATLAB (v.R2018a) for hierarchical clustering analysis using JMP and PCA using MATLAB functions and in-house scripts. For finished products W, X, Y, and Z, the measured *Ginkgo biloba* content per gram was scaled to subtract the amount of filler according to the manufacturer. Respectively, the measured values were multiplied by 5.83, 5.83, 4.17, and 4.17 for finished products W, X, Y, and Z.

A GbE sample lot was considered to be characteristic of GbE when its constituent content substantially matched a known GbE standard, e.g., NIST SRM3247, or when chemometric analysis clustered it with a known GbE standard. A sample lot was uncharacteristic when its constituent content did not match a known GbE standard, when it contained constituents not present in a known standard, or when it did not cluster with known standards after chemometric analysis.

## Results

### Non-targeted HPLC-ELSD

**Fig. 1:**
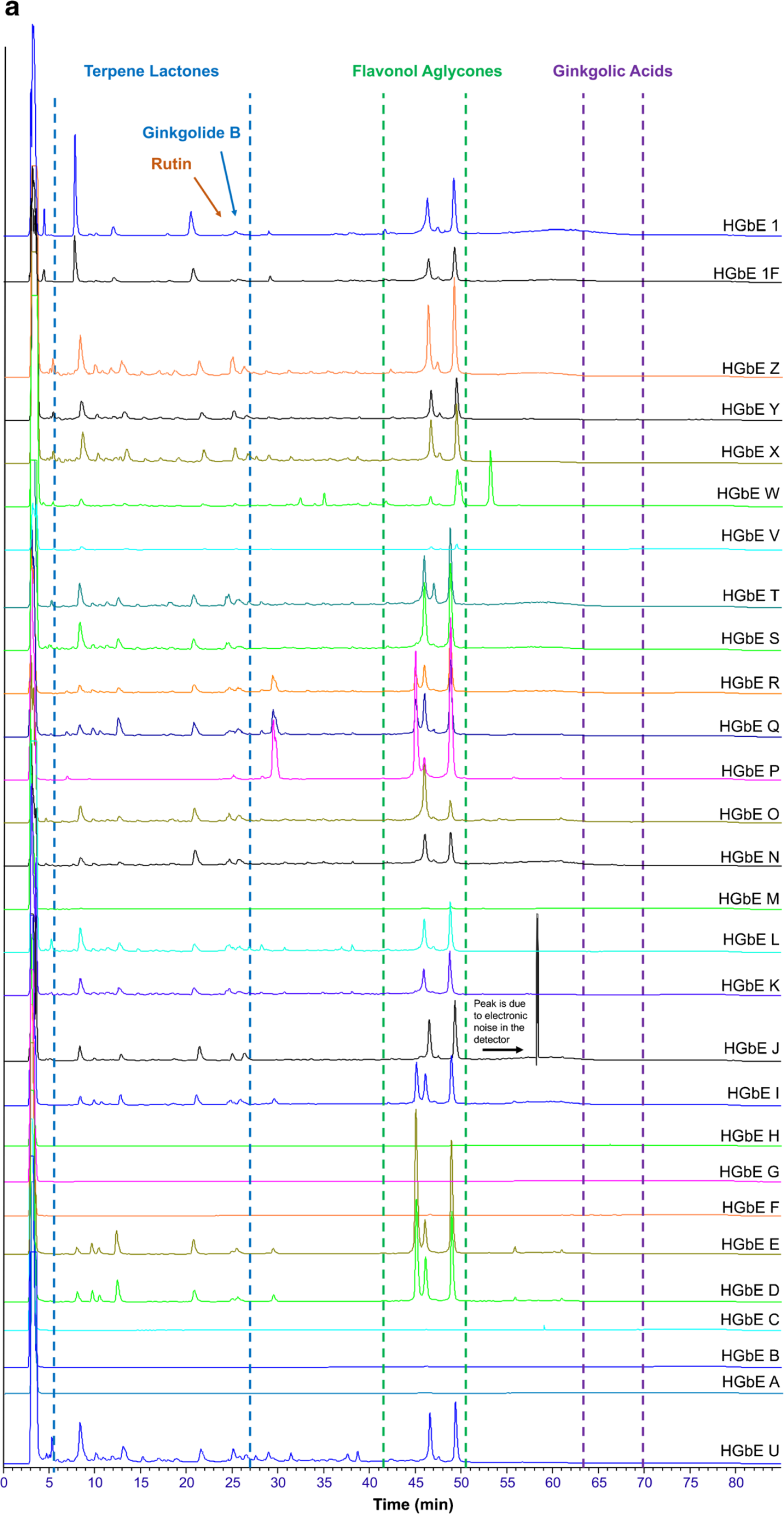
Chromatograms of unhydrolyzed GbE unfinished samples (GbE A–T), commercial GbE finished products (GbE W–Z), NTP lots (1, 1F), and NIST standard extract (GbE U) and tablet (GbE V)

**Fig. 2:**
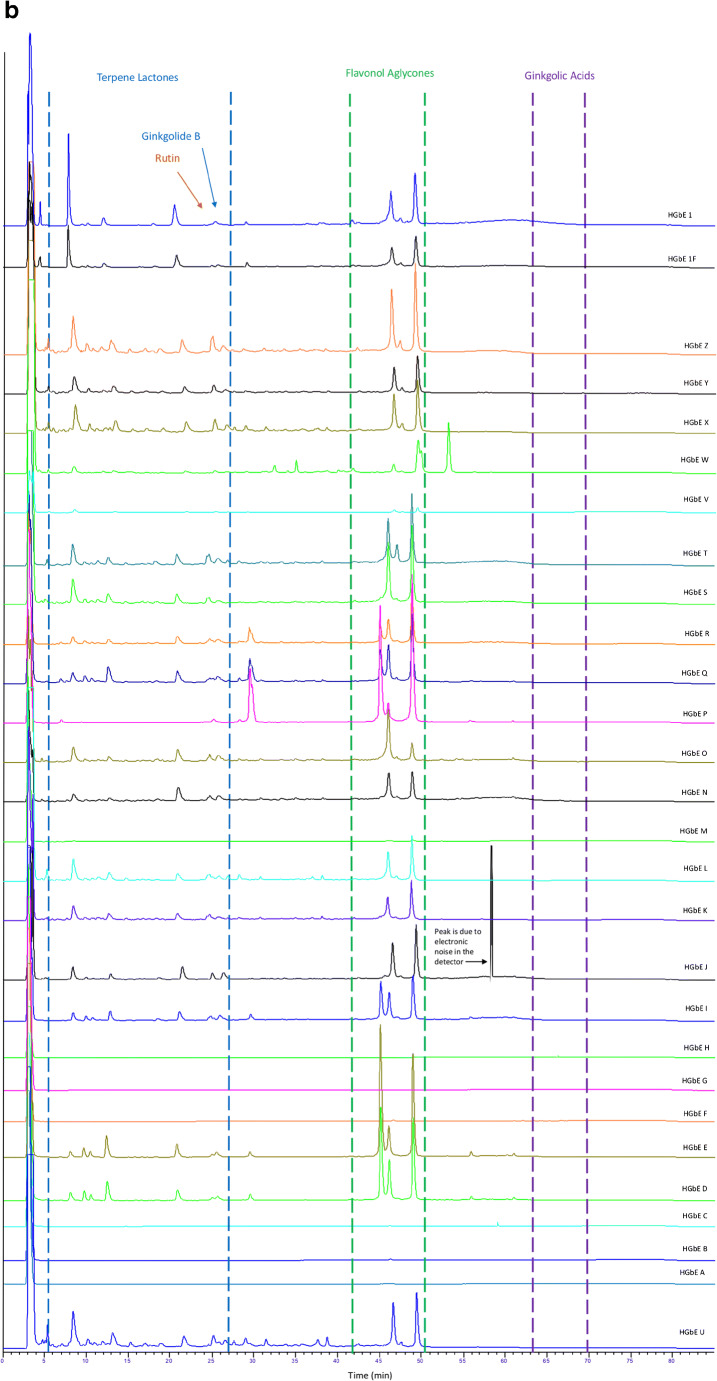
Chromatograms of hydrolyzed GbE unfinished samples (GbE A–T), commercial GbE finished products (GbE W–Z), NTP lots (1, 1F), and NIST standard extract (GbE U) and tablet (GbE V), Peak at ~ 58 min in GbE J chromatogram is due to electronic noise in the detector

**Fig. 3:**
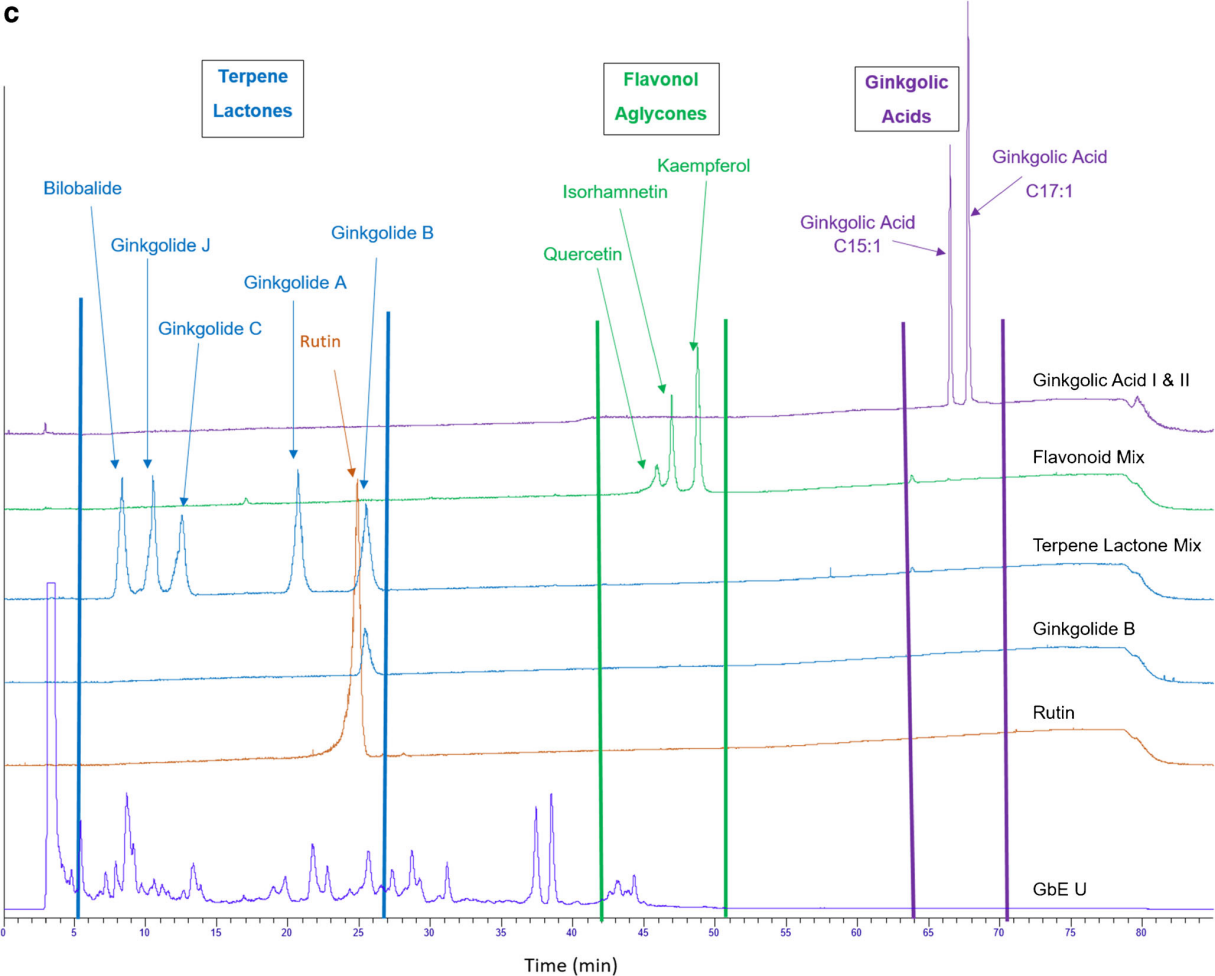
Chromatograms of GbE constituent standards bilobalide, ginkgolide B, ginkgolic acids I and II, GbE flavonol aglycone and terpene trilactone mixtures, and NIST standard extract (GbE U)

GbE constituent standards were used to establish approximate retention time ranges between 0 and 27 min, 42 and 51 min, and 64 and 71 min, for terpene trilactones, flavonol aglycones, and ginkgolic acids, respectively. Flavonol glycosides were estimated to elute between approximately 23 and 43 min, based upon the disappearance of peaks in this region after hydrolysis, and the presence of rutin at ~ 24 min (Fig. 1). Chromatograms from samples were visually compared with the NIST unfinished GbE SRM (3247) and each other on the same X-Y scale for the presence or absence of peaks in each retention time range as well as peak shape and intensity. Figures 1 and 2 show chromatograms for unhydrolyzed and hydrolyzed samples, respectively; Fig. 3 shows the chromatograms for the unhydrolyzed GbE constituent standards and the NIST SRM 3247.

Chromatograms of unhydrolyzed unfinished products (samples A–T, 1, and 1F in Fig. 1) showed wide variability among themselves and versus NIST SRM 3247 (U). Several unfinished products (D, E, I, J, K, N–S) had large peaks corresponding to retention times of flavonols, indicating potential adulteration of these samples via the addition of flavonols. Other unfinished products (A, B, C, F, and G) had few peaks characteristic of NIST SRM 3247 (U) with one sample (G) showing only 2 peaks in the retention time range of ginkgolic acids, and four samples (A, B, C, and F) showing a single peak corresponding to the retention time of rutin. Unfinished products H and M showed no significant peaks over the entire chromatogram. NTP unfinished products 1 and 1F and unfinished products L and T had expected peaks in the retention time range of the terpene trilactones and compared favorably with the NIST SRM 3247 (U) at all retention times.

Unhydrolyzed finished products (samples W–Z in Fig. 1) showed peaks over the expected retention time range of the terpene trilactones and closely matched the NIST SRM 3247 (U). All finished samples (W–Z) in addition to the finished NIST SRM 3248 (V) had late-eluting peaks (in the retention time range of ginkgolic acids, but not corresponding to the retention time of either ginkgolic acid standard), potentially from excipients in the formulated material. As expected, the finished NIST SRM 3248 (V) showed lower peak heights across the chromatogram because the mass of the finished product analyzed could not be adjusted based on GbE content as no value was provided. Sample W showed additional peaks in the suspected flavonol glycoside region (~ 23 to 42 min) and ~ 47 to 54 min, a region that did not correspond to any known GbE constituents or SRM peaks, likely due to the label-declared presence of *Centella asiatica* (commonly known as Gotu kola) in this finished combination product.

After hydrolysis (Fig. 2), during which flavonol glycosides were converted to corresponding flavonols, chromatograms of all unfinished samples except A–C, F, G, H, and M visually resembled the NIST SRM 3247 (U). Chromatograms of hydrolyzed unfinished samples showed expected reductions in peak number and peak size in the suspected flavonol glycoside retention time region and corresponding increases in peak number and/or peak size in the retention time range for flavonol aglycones. Reductions in peak numbers were also seen in the terpene trilactone retention time range, which may correspond to hydrolysis of early eluting glycosides. Hydrolyzed finished products with the exception of sample W, visually resembled the NIST SRM 3247 (U). As discussed above, the finished NIST SRM 3248 (V) showed lower peak heights across the chromatogram. Finished product W retained a peak eluting slightly outside the flavonol retention time region and several small early-eluting peaks also present in the unhydrolyzed material.

In summary, the qualitative analysis of the chromatograms using the known elution time ranges of constituent compounds and comparisons with a SRM could reasonably differentiate sample products in unhydrolyzed samples. Based upon these analyses, unfinished product samples 1 and1F (NTP), and L and T and the finished products X, Y, and Z were most similar to the NIST GbE SRM (U), while unfinished products A, B, C, F, G, H, and M were most dissimilar.

**Fig. 4:**
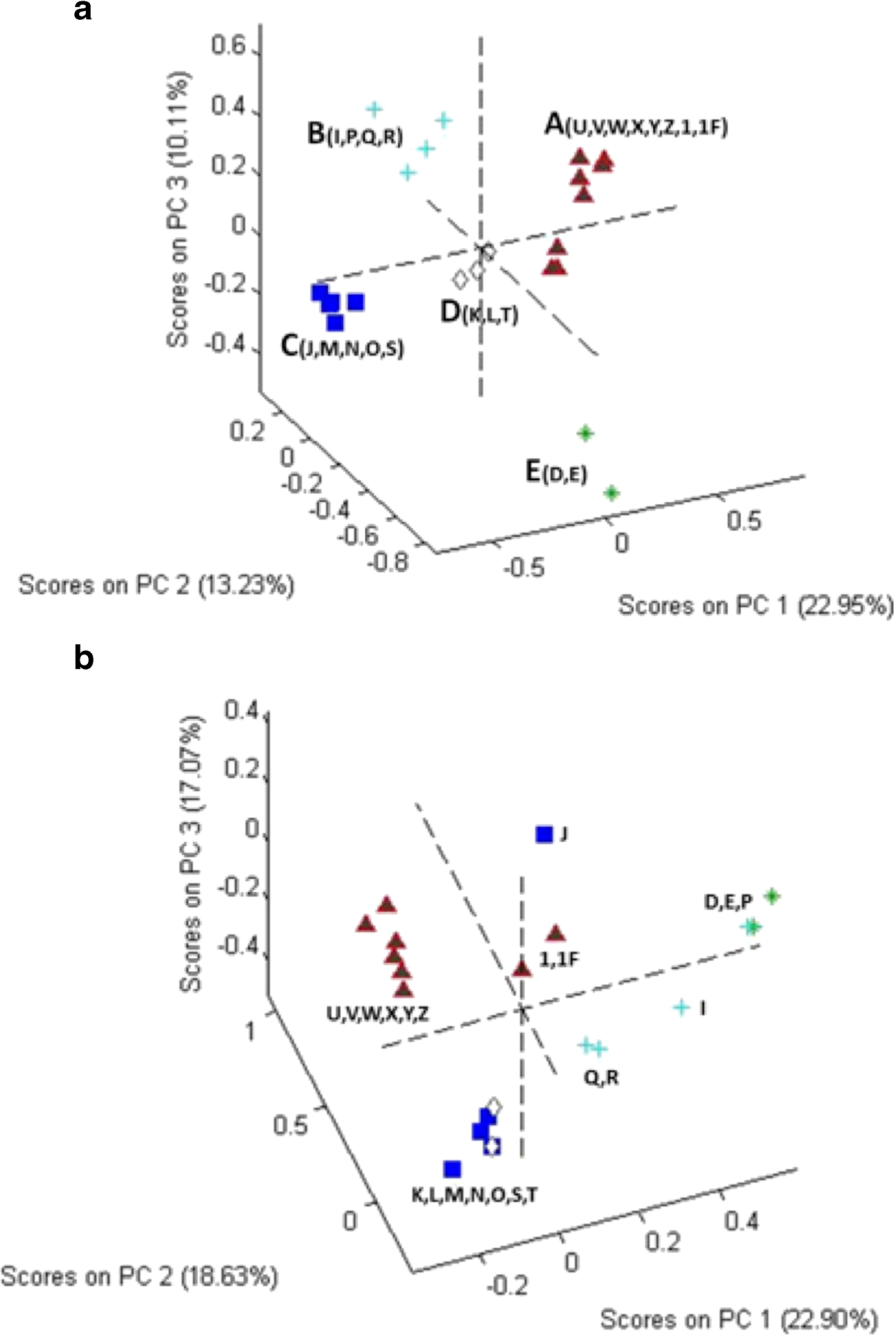
PCA score plots for chromatograms of **a** unhydrolyzed and **b** hydrolyzed samples. In **a**, the clusters are color coded: cluster A (samples U, V, W, X, Y, Z, 1, and 1F), cluster B (samples I, P, Q, and R), cluster C (samples J, M, N, O, and S), cluster D (samples K, L, and T), and cluster E (samples D and E). In **b**, the color codes from **a** are used to illustrate the change in relative composition of the samples

A PCA score plot from chemometric analysis of pre-processed, non-targeted data is shown in Fig. 4a and b for unhydrolyzed and hydrolyzed samples, respectively. Six unfinished products (A, B, C, F, G, and H) were omitted from the analysis because they contained only two or fewer peaks in their chromatograms. The lack of a more complex chromatogram suggested an error in the materials supplied or unique extraction processes. When included in the PCA, they provided scores that were well away from the central tendency and produced a large increase in the total variance of the data set.

Although less than obvious in Fig. 4a, rotation of the plot established that the 20 commercial samples and 2 NIST SRMs fell into 5 clusters. Cluster A was the largest, consisting of 8 samples: from NIST (samples U and V), from commercial Source 16 (finished products W, X, Y, and Z), and from NTP *Ginkgo biloba* unfinished product samples (1 and 1F). The other clusters and their composition were cluster B (unfinished products I, P, Q, and R), cluster C (unfinished products J, M, N, O, and S), cluster D (unfinished products K, L, and T), and cluster E (unfinished products D and E). Comparison of chromatograms (Fig. 1) with PCA plots (Fig. 4a) illustrates the effects of data processing. Whereas sample M looks very different from other cluster C samples in the chromatogram (Fig. 1), normalization of the data allows for better peak detection and reveals that based on peak presence (as opposed to peak height), unfinished product M appeared similar to unfinished products J, N, O, and S.

Figure 4b shows the impact of hydrolysis on the sample composition. The samples retain the icons and colors used to identify them in Fig. 4a, but their positions have shifted. For cluster A, the 2 NTP unfinished product samples (1 and 1F) have moved further away from the other samples, closer to the origin of the plot. With the exception of unfinished product J, clusters C and D have merged. Unfinished product P from cluster B joined cluster E and the remaining 3 are separate from the other samples but are not clearly together. Figs. S1–S4 (see ESM) present a series of truncated, derivatized, and normalized chromatograms (see “Methods” section) that illustrate how the components change with hydrolysis and provide a basis of comparison of the different samples. These chromatograms (using every point) were the basis for the PCA plots shown in Fig. 4a and b.

### Non-targeted NMR

**Fig. 5:**
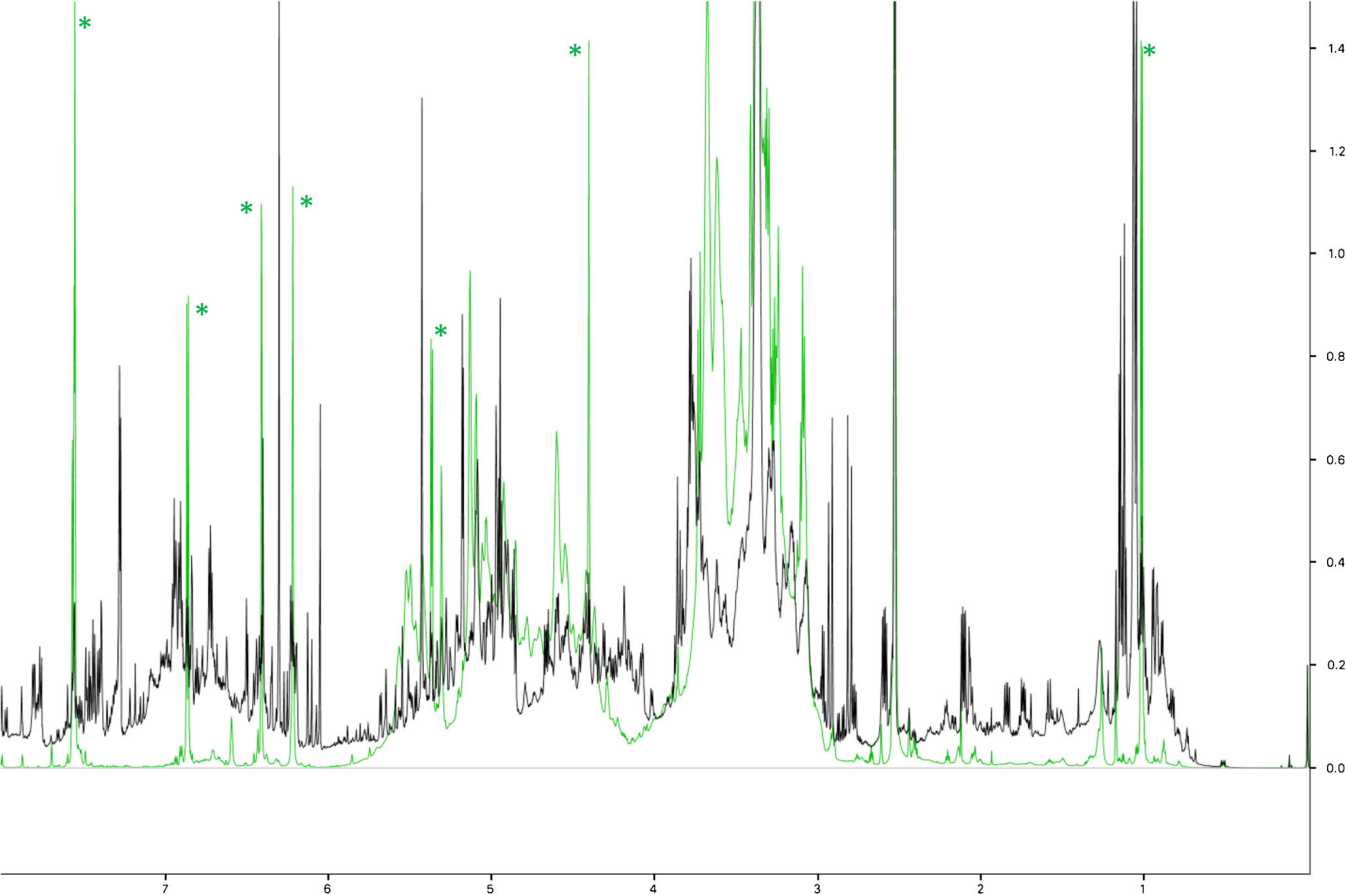
An overlay of the NMR spectra of GbE U (NIST standard, black line) versus GbE A (green line). Green asterisks indicate rutin peaks

**Fig. 6:**
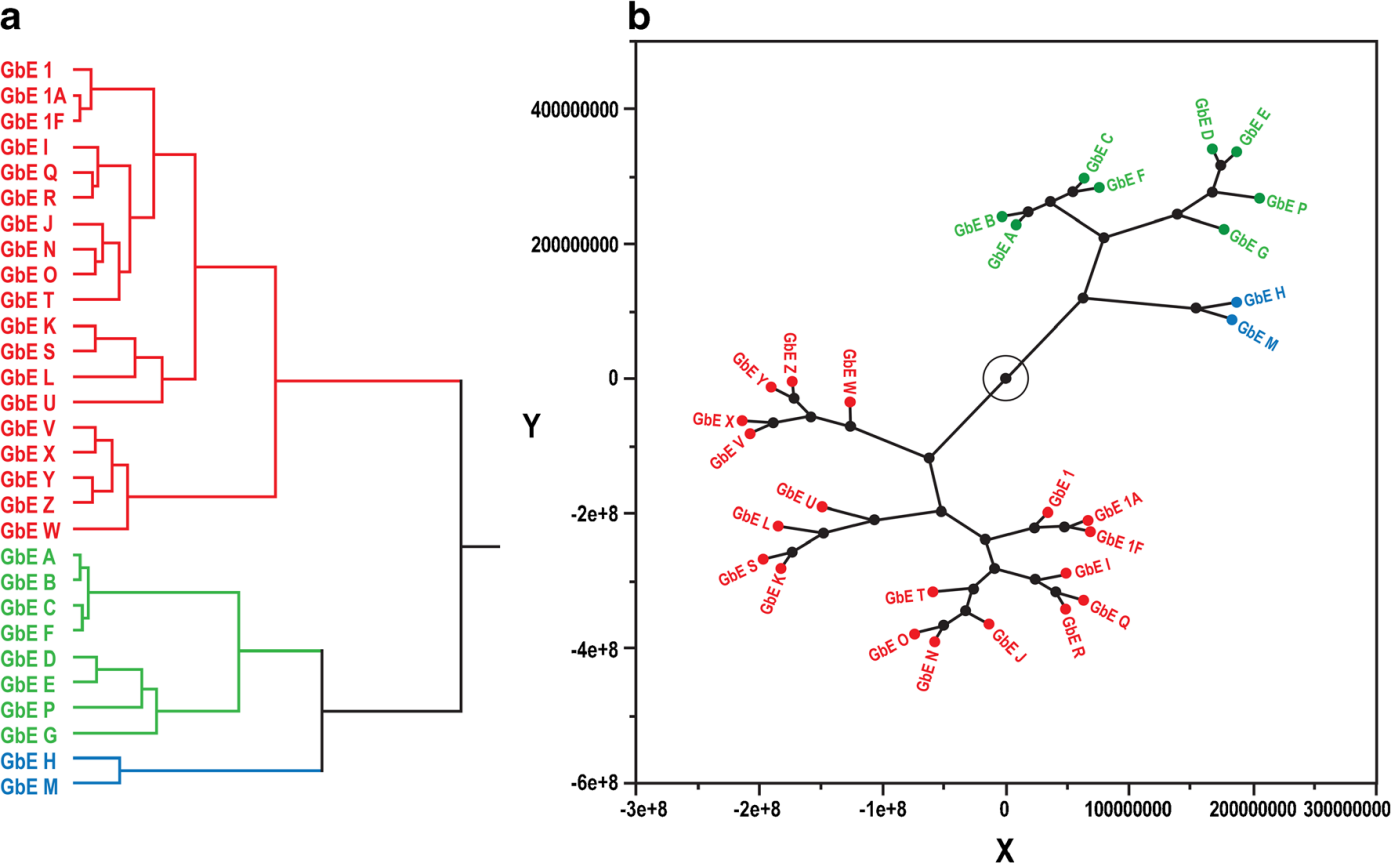
Unsupervised analysis of the NMR spectra of GbE. **a** Dendrogram analysis of the similarities of NMR spectra. **b** Constellation plot to visualize the hierarchical clustering results

Similar to the comparisons above, NMR spectra of the sample extracts can be qualitatively compared. Figure 5 shows a comparison of the NIST standard (U) with unfinished product A that was identified above as having very few components. Differences in the peak frequencies and intensities are immediately apparent in the aromatic region (6–8 ppm) and in the aliphatic region, especially 1–3 ppm. It was hypothesized that an unsupervised analysis of the frequency and intensity data could readily differentiate the various samples. Figure 6 shows hierarchical clustering as a dendrogram (a) and a constellation plot (b) demonstrating the relationships in phytochemical composition among samples. All three of the unfinished GbE product samples from the NTP test article (1, 1A, and 1F) are closely clustered, indicating that chemical composition was not significantly altered by the different storage conditions. Other unfinished products that cluster with the NTP test article consist of samples I, J, K, L, N, O, Q, R, S, and T and the NIST SRM (U). As in the non-targeted HPLC-ELSD analysis, unfinished GbE products with very low levels of flavonols and terpene trilactones cluster together: A, B, C, D, E, F, G, and P cluster in one group, which is close to the group of unfinished products containing samples H and M. Unlike in the HPLC-ELSD analysis, the mass of the finished products was not adjusted based on achieving an equivalent level of GbE to that in the unfinished products. Therefore, even though finished products W–Z and the NIST finished product SRM (V) are in the same cluster as many of the unfinished products including the NIST unfinished product SRM (U), a direct comparison between the finished and unfinished products cannot be made.

### High-performance thin-layer chromatography

**Fig. 7:**
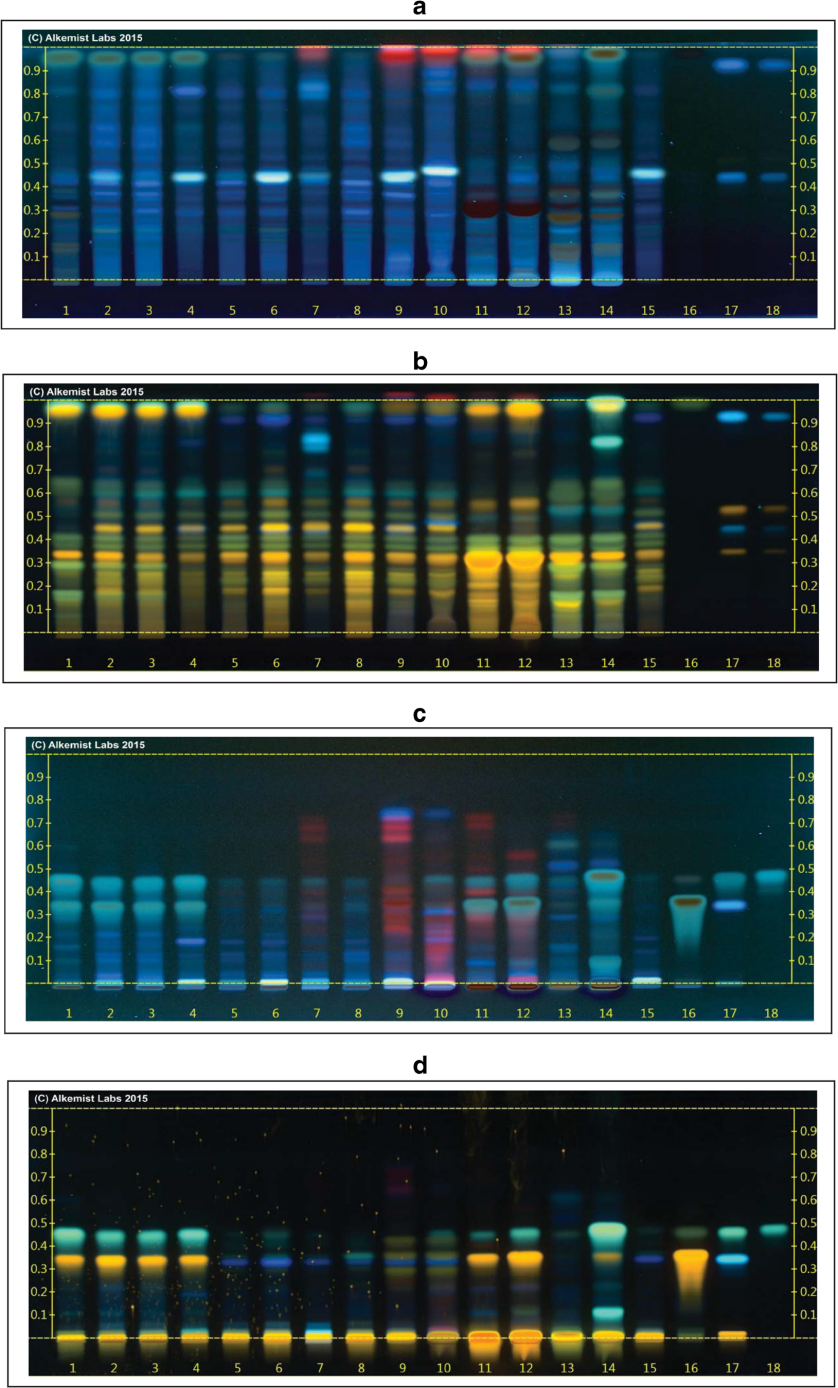
High-performance thin layer chromatogram showing: (**a**) GbE P, Q, R, S, T, U, W, and 1A (Lanes 1–8); Ginkgo biloba leaf (Lanes 9 and 10); Sophora japonica flower (Lanes 11 and 12), S. japonica fruit (Lanes 13 and 14); NIST standard GbE SRM 3247 (Lane 15); genistein (Lane 16); cffeic acid, hyperoside, rutin, and chlorogenic acid (Lanes 17 and 18). Mobile phase (System 1): ethyl acetate: acetic acid: formic acid: water (10/1.1/1.1/2.6) @35–40% humidity. Visualized at 365 nm (b) Lane assignments same as in (**a**). Visualized with Natural Product Reagent + polyethylene glycol @365 nm. (**c**) Lane assignments for 1–15 same as in (**a**) except quercetin, genistin, and genistein (Lane 16); isorhamnetin, caffeic acid, rutin, hyperoside, chlorogenic acid (Lane 17), kaempferol (Lane 18). Mobile phase (System 2): toluene:ethyl acetate: formic acid: (7/3/1) @35–40% humidity. Visualized at 365 nm. (**d**) Lane assignments as in (**c**). Visualized with Natural Product Reagent + Polyethylene glycol @365 nm

HPTLC was used to analyze 17 GbE samples including 14 unfinished GbE product samples (D, E, I, J–L, N–T), one unfinished *G. biloba* leaf powder sample (G), one finished GbE product (W), and the NIST unfinished GbE SRM (U) from 16 unique suppliers against known *G. biloba* extract and leaf standards and standards of known adulterants (e.g., *Sophora japonica*) using the two analytical systems described above. Results of the analysis of each sample were reported as characteristic or uncharacteristic of *G. biloba* extract. Chromatograms from unfinished samples P–U are shown in lanes 1–6 of Fig. 7, while those from finished sample W and the NTP test article (1A) are shown in lanes 7 and 8, respectively. Panels a and c in Fig. 7 show visualization at 365 nm after separation via system 1 (panel a) or system 2 (panel c), respectively. Panels b and d show visualization at 365 nm using Natural Product Reagent™ and polyethylene glycol after separation via system 1 (panel b) or system 2 (panel d), respectively. Three of the 15 submitted unfinished product samples (L, T, 1A) and the NIST unfinished GbE SRM (U) were found to have features characteristic of GbE, while twelve unfinished products (D, E, G, I, J, K, N, and O–S) and one finished product (W) were found to be uncharacteristic of GbE. Uncharacteristic samples fell into broad categories: samples containing bands consistent with the presence of a different plant species and/or samples containing bands corresponding to the presence of added flavonol aglycones.

Unfinished product sample S (Fig. 7, lane 4) is an example of a sample found not to be characteristic of GbE due primarily to the presence of bands indicating the presence of non-*Ginkgo* plant species.

Using system 1, visualized at 365 nm, unfinished sample S (Fig. 7a, lane 4) displayed a bright band corresponding to the single band at *R*_f_ ~ 0.45 present in a NIST standard GbE (lane 15) and samples of *G. biloba* leaf (Fig. 7a, lanes 9 and 10), but contained an additional diffuse band at *R*_f_ ~ 0.90 not present in the NIST standard extract, but consistent with bands seen in *Sophora japonica* (flower) (Fig. 7a, lanes 11 and 12). When visualized using NPR + PEG, unfinished sample S (Fig. 7b, lane 4) showed a single band at *R*_f_ ~ 0.35, a split band at *R*_f_ ~ 0.40, and a band at *R*_f_ ~ 0.45 that were also present in the NIST standard (Fig. 7b, lane 15) but were fainter than those present in *G. biloba* leaf standards (Fig. 7b, lanes 9 and 10). The single band seen in unfinished sample S at *R*_f_ ~ 0.60 was also present in the *S. japonica* (flower and berry) samples (Fig. 7b, lanes 11–14) but was absent from the NIST GbE standard. Bright bands present in unfinished sample S at *R*_f_ values of > ~ 0.90 were not seen in the NIST GbE standard but were present in all but one of the *S. japonica* samples.

Using system 2 visualized at 365 nm with or without NPR + PEG, unfinished sample S (Fig. 7c and d, lane 4) shows two prominent bands at *R*_f_ values of 0.35 and 0.45 that were not seen in the NIST unfinished GbE SRM (Fig. 7c and d, lane 15) but are characteristic bands of the flavonol aglycones quercetin, isorhamnetin, and kaempferol (Fig. 7c, lanes 16–18). A bright band at the origin seen in unfinished sample S (Fig. 7d, lane 4) corresponds to a bright band at the origin, seen in the NIST standard GbE (Fig. 7d, lane 15) and the *G. biloba* leaf (Fig. 7d, lanes 9 and 10), but this band is also present in the *S. japonica* samples. Based on these results, unfinished sample S may be a mixture of *S. japonica* (flowers and berries) and GbE with added flavonol aglycones.

The NTP unfinished GbE test article (1A; NTP; Fig. 7a, lane 8) is an example of a sample found to be characteristic of GbE. Bands seen using system 1 visualized at 365 nm matched those of the NIST standard extract (Fig. 7a, lane 15). A faint band present at *R*_f_ ~ 0.40 matched a brighter band at the same *R*_f_ in the NIST standard extract. No extraneous bands were observed. A bright band seen in *G. biloba* leaf at *R*_f_ > ~ 0.90 (Fig. 7a, lanes 9–10) was not present in the NTP test article (1A), but this band was also missing from the NIST standard extract (Fig. 7a, lane 15). When visualized at 365 nm with NPR + PEG, a pattern of bands was seen in the NTP test article (1A; Fig. 7a, lane 8) that was also seen in the NIST standard extract (Fig. 7a, lane 15). Bands present in the NIST standard extract at *R*_f_ values of ~ 0.35, 0.45, and 0.65 are also seen in the NTP test article (1A). A double band at *R*_f_ ~ 0.40 in the NIST standard is present in the NTP test article (1A). Extraneous bands that would indicate the presence of non-GbE species were not present.

Using system 2 visualized at 365 nm with or without NPR + PEG, a single faint band at an *R*_f_ of ~ 0.35 seen in the NTP test article (1A; Fig. 7c and d, lane 8) matched the NIST standard extract, but had slightly higher intensity (Fig. 7c and d, lane 15). Notably, unhydrolyzed NTP test article (1A) did not express bands corresponding to quercetin, genistin, or genistein (Fig. 7c and d, lane 16), isorhamnetin, caffeic acid, rutin, hyperoside, or chlorogenic acid (Fig. 7c and d, lane 17), or kaempferol (Fig. 7c and d, lane 18), which would have indicated possible adulteration. Bands associated with other non-*Ginkgo* species were also absent.

### Quantitation of GbE constituents by HPLC

Targeted quantitation of GbE constituents was performed on unhydrolyzed and hydrolyzed samples from 20 commercially available unfinished GbE or *G. biloba* leaf powder products (A–T); 4 finished GbE products (W–Z); the unfinished NTP test article (1, 1A, and 1F); and the NIST unfinished extract SRM (U) and finished tablet (V). The weight percent of terpene trilactones (bilobalide and ginkgolides A, B, C, and J); rutin; flavonol aglycones (quercetin, kaempferol, and isorhamnetin); GA-I and GA-II; and ginkgotoxin was determined for each unhydrolyzed (Table 1) or hydrolyzed (ESM Tables S3 and S4) GbE sample. Correlation coefficients for constituent standard curves run with the samples were > 0.99 for all constituents (ESM Table S2). Constituent concentrations in duplicate samples of the NIST unfinished GbE SRM were compared with NIST’s reported values and indicated good agreement between the replicates and the reported values for most constituents (ESM Table S5).

**Table 1:** Percent by weight of GbE constituents in unhydrolyzed samples quantitated by HPLC-ELSD/UV

Source/type	GbE ID	Terpene trilactones^1^					Rutin	Flavonols^1^		
		Bilobalide	A	B	C	J		Quercetin	Kaempferol	Isorhamnetin
NTP UFP	1	6.30	5.66	1.75	2.21	*1.19*	3.45	*0.49*	*0.15*	*0.05*
NTP UFP	1A	7.33	5.18	1.89	1.99	1.70	3.95	*0.48*	*0.16*	*0.08*
NTP UFP	1F	6.64	5.67	1.72	2.12	*1.05*	3.58	*0.52*	*0.15*	*0.04*
1 UFP	A	ND	ND	ND	ND	ND	3.40	*0.15*	ND	ND
1 UFP	B	ND	ND	ND	ND	ND	2.60	*0.14*	ND	ND
1 UFP	C	ND	ND	ND	ND	ND	1.92	*0.11*	ND	ND
2 UFP	D	1.48	2.09	1.42	2.77	*1.36*	1.54	4.22	0.95	*0.19*
3 UFP	E	*1.31*	2.30	1.57	2.80	*1.39*	1.40	3.46	1.59	*0.18*
4 UFP	F	ND	ND	ND	ND	ND	2.22	*0.15*	ND	ND
5 UFP	G	ND	*0.62*	*0.77*	*0.83*	*0.65*	ND	ND	ND	ND
5 UFP	H	ND	ND	ND	ND	ND	ND	ND	ND	ND
5 UFP	I	1.53	2.63	1.41	1.89	*1.19*	1.05	5.17	1.17	*0.23*
6 UFP	J	2.29	3.30	1.76	1.68	*1.14*	0.57	5.08	3.78	*0.27*
7 UFP	K	3.13	2.78	1.30	1.92	*1.27*	1.79	3.48	2.43	*0.21*
8 UFP	L	3.02	3.25	1.29	1.63	*1.15*	4.16	*0.45*	*0.36*	*0.19*
9 UFP	M	*1.31*	ND	ND	ND	ND	*0.09*	*0.44*	*0.31*	*0.11*
9 UFP	N	1.70	4.41	1.91	*1.18*	*0.88*	0.91	4.51	2.33	*0.32*
10 UFP	O	2.75	3.78	1.82	1.72	*1.27*	1.25	6.36	*0.68*	*0.22*
11 UFP	P	*1.07*	ND	*0.64*	*0.75*	*1.39*	2.12	1.50	2.34	*0.17*
12 UFP	Q	1.95	3.18	1.57	2.46	*1.48*	1.79	2.91	1.02	*0.20*
13 UFP	R	2.09	2.75	1.51	1.56	*1.32*	0.97	2.42	1.25	*0.23*
14 UFP	S	2.95	2.24	*1.11*	1.62	*1.26*	0.88	4.07	3.47	*0.30*
15 UFP	T	2.29	3.53	1.47	1.43	*1.07*	4.03	*0.36*	*0.48*	*0.26*
NIST SRM	U	3.53	2.79	2.74	1.66	2.03	3.04	*0.36*	*0.29*	*0.18*
NIST SRM	V	*0.97*	*1.03*	*0.67*	*0.82*	ND	0.49	*0.21*	ND	ND
16 FP	W	4.19	6.11	2.91	1.76	*1.16*	4.56	0.71	*0.63*	*0.06*
16 FP	X	3.73	4.76	1.51	2.12	1.69	4.47	*0.33*	*0.16*	*0.05*
16 FP	Y	3.77	4.45	1.38	1.96	1.58	4.15	*0.37*	*0.21*	*0.05*
16 FP	Z	3.42	4.48	1.28	1.97	*1.54*	4.21	*0.30*	*0.18*	*0.05*

^1^Values reported for quercetin, kaempferol, and isorhamnetin are aglycone concentrations. Glycoside values may be obtained by multiplying the quercetin, kaempferol, and isorhamnetin values by 2.504, 2.588, and 2.437, respectively

Values in italics are below LOQ. See Table S2 for LOD and LOQ values for each GbE constituent. *ND*, not detected

^*2*^*FP*, finished product; *UFP*, unfinished product; *SRM*, standard reference material

Terpene trilactone constituents were measured in most of the GbE samples tested, with the exception of unfinished products A, B, C, F, G, and H, which showed few if any peaks for any of the GbE constituents. In unhydrolyzed samples, total terpene trilactone content ranged from non-detectable (no peaks detected above baseline noise) to 1.3 to 18.5% in GbE samples with detectable constituent peaks for the target substances (Table 1). Of the terpene trilactones present, bilobalide and ginkgolide A had the highest average concentrations (3.2 and 3.5% w/w, respectively). In hydrolyzed samples, total terpene trilactone concentrations were relatively unchanged, ranging from 1.2 to 20.0%, with bilobalide and ginkgolide A remaining as the largest constituents. Unfinished product samples 1, 1A, and1F from the NTP unfinished GbE product were most similar to the other characteristic unfinished products (L and T; Table 1) but had somewhat higher unhydrolyzed total terpene trilactone content than the NIST unfinished GbE SRM 3247 or the other characteristic finished and unfinished samples (17.6% vs. 11.4% and 14.2%, respectively). The difference was driven by higher mean values for bilobalide and ginkgolide A, although the pattern of bilobalide > ginkgolide A > ginkgolide B > ginkgolide C > ginkgolide J seen in the NIST standards and characteristic samples was the same for NTP unfinished GbE samples (1, 1A, and 1F).

Constituent flavonols ranging from 0.1 to 6.4% were seen in the targeted analysis for all but 2 of the unhydrolyzed GbE finished and unfinished samples. Unhydrolyzed GbE samples with detectable constituent peaks for the targeted constituents had total flavonol content ranging from 0.7 to 9.1% w/w. Quercetin was the largest aglycone measured across all samples with values ranging from 0.3 to 6.4%. Concentrations of flavonol aglycones in unhydrolyzed GbE samples should be very low and their presence at relatively high concentrations in some samples is indicative of adulteration of the sample with added aglycone constituents to mimic the content in hydrolyzed standardized GbE. After hydrolysis, flavonol content rose as expected, ranging from 2.8 to 11.8%. After conversion to corresponding glycoside values, mean total flavonol glycoside content of hydrolyzed GbE samples was 23.7 ± 6.1% and was similar to the value seen in finished products (24.9 ± 1.3%) and the NIST standard (25.0 ± 1.2%). Total aglycone content along with individual flavonol contents of the unhydrolyzed NTP samples was most similar to the NIST unfinished GbE SRM, and finished products (0.71% vs. 0.83%, and 0.78% w/w, respectively). After hydrolysis, mean flavonol content in NTP samples, measured as glycosides, was 28.0 ± 0.9% w/w, similar to the means of the NIST unfinished GbE SRM, and finished products (24.9 ± 1.3% and 25.0 ± 1.2% w/w, respectively). Interestingly, after hydrolysis, all of the unfinished GbE products, NTP unfinished GbE samples, finished products, and the NIST unfinished GbE SRM had similar flavonol glycoside content.

Small amounts of GA-I and GA-II were found in most samples, but values for total ginkgolic acids were < 0.005% in all but 2 samples. The two samples with high total ginkgolic acids were unfinished products G and N, which had values of 0.3 and 0.1% w/w, respectively. In general, unfinished GbE samples had mean total ginkgolic acid concentrations that were higher (0.048 ± 0.033) than the mean values (ND and 0.00028 ± 0.00006% w/w, seen in finished products and the NIST unfinished SRM, respectively). Total ginkgolic acid values in the NTP unfinished GbE product samples (1, 1A, and 1F) were low, with a mean value of 0.0018 ± 0.0002% w/w, which was lower than the mean for other unfinished GbE samples (0.048%).

Ginkgotoxin levels were generally low, with individual values < 0.1% in all but one sample before or after hydrolysis. Mean values in all unfinished GbE samples and the NIST unfinished GbE SRM were similar (0.03–0.05% w/w), but were higher than finished products (0.01%) or the unfinished NTP GbE samples (0.01%).

### Quantitation of GbE constituents by NMR

Targeted analysis of unhydrolyzed finished and unfinished GbE samples by NMR was conducted using prepared constituent standards of bilobalide; ginkgolides A, B, C, and J; rutin; quercetin; kaempferol; and isorhamnetin. These constituents were used to create a standard compound library for spectral comparisons. The NMR shifts of the constituent standards matched well with published spectra [20]. Fig. S5 (see ESM) shows the NMR data for the NIST unfinished GbE SRM (U) and indicates that many compounds are apparent in the data (black line), but only the standard compounds were quantified, if present (red lines). Fig. S6 (see ESM) zooms in on the region from 1.5 to 2.2 ppm to demonstrate that despite the number of other compounds, characteristic peaks of the markers could be identified, and the intensity measured for comparison with the DSS reference compound (not shown).

The measured concentrations were converted to percent by weight (g/g; Table 2) for direct comparison with the targeted HPLC constituent results below, shown in ESM Fig. S7. The correlation coefficient displayed below each graph shows that the correlations were strongly positive and generally better when there were greater amounts of the constituent compounds present.

**Table 2:** Percent by weight of unhydrolyzed GbE constituents quantitated by NMR

Source/type^2^	GbE ID	Terpene trilactones					Rutin	Flavonols^1^		
		Bilobalide	A	B	C	J		Quercetin	Kaempferol	Isorhamnetin
NTP UFP	1A	3.10	3.30	0.70	0.60	0.30	1.90	ND	ND	ND
NTP UFP	1F	9.80	10.60	1.80	2.10	1.00	7.40	ND	ND	ND
NTP UFP	1	10.30	8.60	1.60	1.90	0.90	6.50	ND	ND	ND
1 UFP	A	ND	ND	ND	ND	ND	8.80	0.10	ND	ND
1 UFP	B	ND	ND	ND	ND	ND	13.10	ND	ND	ND
1 UFP	C	ND	ND	ND	ND	ND	15.60	ND	ND	ND
2 UFP	D	0.90	2.20	0.70	1.50	0.70	1.90	2.50	1.00	ND
3 UFP	E	0.50	1.80	0.50	1.10	0.50	1.90	1.60	1.00	ND
4 UFP	F	ND	ND	ND	ND	ND	18.10	ND	ND	ND
5 UFP	G	ND	ND	ND	ND	ND	ND	ND	ND	ND
5 UFP	H	ND	ND	ND	ND	ND	ND	ND	ND	ND
5 UFP	I	0.50	1.10	0.40	0.50	0.30	ND	1.60	0.50	ND
6 UFP	J	1.10	2.10	0.60	0.50	0.30	ND	1.80	1.30	ND
7 UFP	K	3.90	3.50	1.10	1.50	0.90	2.70	3.70	2.70	ND
8 UFP	L	3.90	3.00	1.00	1.30	0.80	5.70	ND	ND	ND
9 UFP	M	0.60	0.60	0.20	0.20	0.10	ND	1.00	0.70	ND
9 UFP	N	1.50	4.30	1.40	0.50	0.40	ND	4.30	1.90	ND
10 UFP	O	2.90	4.50	1.40	1.00	0.70	ND	8.00	0.90	ND
11 UFP	P	ND	ND	ND	ND	ND	1.40	0.70	1.70	ND
12 UFP	Q	1.20	2.50	0.80	1.40	0.70	2.20	2.10	1.30	ND
13 UFP	R	0.80	1.60	0.60	0.50	0.30	1.30	1.10	0.80	ND
14 UFP	S	0.90	0.80	0.20	0.30	0.20	ND	1.20	0.90	ND
15 UFP	T	2.20	2.30	0.80	0.70	0.40	3.60	0.20	0.30	ND
NIST SRM	U	2.80	2.40	0.60	1.10	0.70	3.00	ND	0.20	ND
NIST SRM	V	0.90	0.70	0.20	0.30	0.20	0.90	ND	ND	ND
16 FP	W	1.90	1.50	0.60	0.80	0.30	0.40	ND	ND	ND
16 FP	X	5.83	5.25	1.75	2.33	1.17	6.42	ND	0.58	ND
16 FP	Y	4.58	4.58	1.25	1.67	1.25	4.58	ND	ND	ND
16 FP	Z	6.25	5.83	1.67	2.08	1.67	6.67	ND	ND	ND

^1^Values reported for quercetin, kaempferol, and isorhamnetin are aglycone concentrations. Glycoside values may be obtained by multiplying the quercetin, kaempferol, and isorhamnetin values by 2.504, 2.588, and 2.437, respectively. *ND*, not detected

^2^*FP*, finished product; *UFP*, unfinished product; *SRM*, standard reference material

**Fig. 8:**
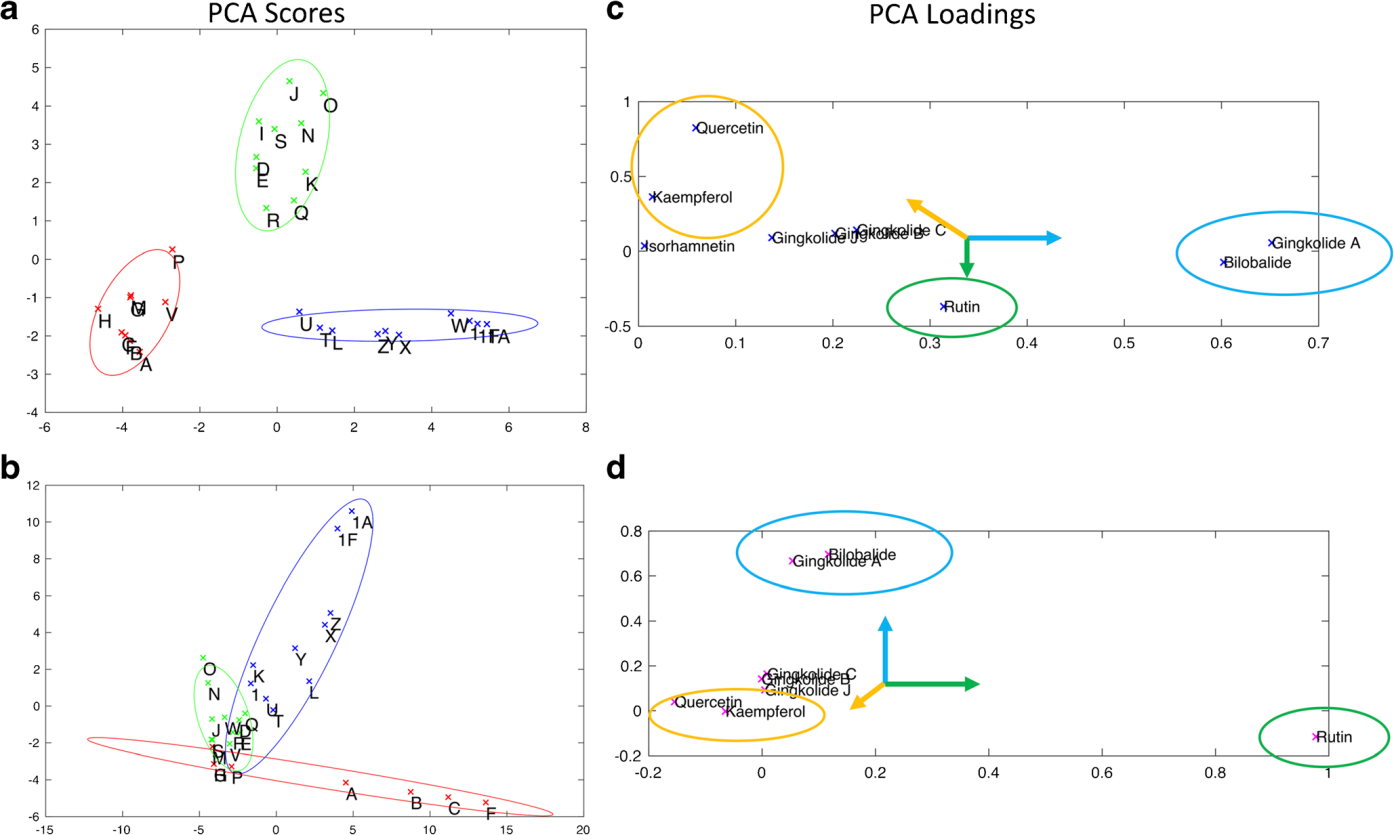
PCA analyses of measured constituents. Panels **a** and **b** show the scores plot of the first 2 principal component analysis of the concentration of the measured constituents by HPLC and NMR, respectively. Panels **c** and **d** show the loading plots from the concentration of the measured constituents by HPLC and NMR, respectively. 95% confidence intervals are shown with ellipses based on the color groups in panels **a** and **b**

To simplify the comparison of the NMR versus the HPLC measurements, PCA plots were created using the measured compounds as inputs. Figure 8 shows the PCA scores (a and b) and loadings (c and d) of the primary components for the HPLC measurements (a and c) versus the NMR measurements (b and d). The first components account for 88% and 92% of the total variance in the HPLC and NMR data, respectively. The samples were grouped by a combination of *K*-means clustering and manual inspection. The NMR clustering was harder to interpret and was based on the PCA plot, as well as the concentration of ginkgolides (Table 2). Subsequently, 95% confidence intervals were calculated based on the clustering.

## Discussion

Evaluating the composition of complex mixtures, such as botanical dietary supplements, is both challenging and essential for selecting test articles for study and interpreting efficacy and/or toxicity data [21]. Case studies comparing across multiple products using available analytical techniques, such as the current GbE example and those presented elsewhere [22, 23], provide valuable information for decision-making and data interpretation. In this paper, we have presented the results of several targeted and non-targeted methods of comparing botanical extracts for 20 commercially available unfinished GbE products, 4 finished products containing a standardized GbE, EGb761®, and the test article used for NTP studies. The results for each sample from each method were compared with each other, and a NIST unfinished GbE SRM (SRM 3247) to assess similarities and differences between these products and to assess their similarity to the NTP unfinished GbE product. Classifications for each of the unfinished products as “characteristic of GbE” or “uncharacteristic of GbE” or “intermediate” are presented in Table 3.

**Table 3:** Categorization of *Gingko biloba* commercial unfinished products via different analyses

Method of analysis	Characteristic GbE samples	Intermediate	Uncharacteristic GbE samples
HPLC-ELSD chromatographic visual inspection	1, 1F, L, T, U^1^	D, E, I, J, K, N, O, P, Q, R, S	A, B, C, F, G, H, M
HPLC-ELSD PCA	1, 1F, U^1^	D, E, I, J, K, L, M, N, O, P, Q, R, S, T	A, B, C, F, G, H
NMR qualitative/unsupervised	1, 1A, 1F, I, J, K, L, N, O, Q, R, S, T, U^1^		A, B, C, D, E, F, G, H, M, P
HPTLC	1A, L, T		D, E, G, I, J, K, N, O, P, Q, R, S
HPLC analysis of GbE marker constituents	1, 1A, 1F, L, T, U^1^	D, E, I, J, K, N, O, Q, R, S	A, B, C, F, G, H, M, P
NMR quantification	1, 1A, 1F, K, L, U^1^, T	D, E, I, J, N, O, Q, R, S	A, B, C, F G, H, M, P

^1^NIST unfinished GbE SRM 3247

The non-targeted methods, both HPLC and NMR, were clearly able to differentiate samples deemed characteristic of GbE versus those deemed uncharacteristic of GbE based on comparison with authentic GbE from known sources. This could be seen in the direct data comparisons (Figs. 1 and 5) and the principal component analyses or the hierarchical clustering (Figs. 4 and 6). The characteristic and uncharacteristic samples generally clustered together. This approach of using global constituent evaluation (i.e., non-targeted chemical analysis) combined with clustering methods has been successfully employed in the literature to identify adulterated GbE [9] and goldenseal [24] samples, as well as in selecting a green tea test article for study [25]. However, as the variance in the data set can change when additional new measurements are brought in for comparison, the principal components can change. So, while this is helpful for observations of clustering within one data set, it is also likely that a visual inspection of the data in Figs. 1 and 5 will be warranted for making comparisons with new botanical samples.

The use of HPTLC methods in assessment of botanical quality has been advocated by the US Pharmacopeia, which suggests that HPTLC provides a complementary procedure to HPLC methods [26]. In this work, HPTLC was able to clearly distinguish between characteristic and uncharacteristic unfinished product samples based on the presence or absence of bands corresponding to known authentic GbE standards and known GbE adulterants. This is shown in Fig. 7 by comparing lanes for each sample (lanes 1–8) with lanes for authentic GbE (lanes 9, 10, and 15) and potential adulterants (lanes 11–14, 16–18). The samples determined to be uncharacteristic of GbE appeared to be composed of small amounts of GbE mixed with a combination of individual constituents and other plant species (e.g., *Sophora japonica*). Samples identified as uncharacteristic via HPTLC were also flagged using the non-targeted HPLC and NMR methods.

Much of the previous work surveying finished and unfinished GbE products across the marketplace used targeted analysis, in effect, quantification of marker constituents with a focus on a subset of terpene trilactones (bilobalide, ginkgolides A and B), flavonol aglycones (quercetin, kaempferol, isorhamnetin), and ginkgolic acids [4, 5, 27]. In the current work, we extended the targeted quantitative analysis to include additional constituents such as ginkgolide J and ginkgotoxin. In looking at the targeted comparisons, the measurements of GbE constituents from both HPLC and NMR were highly correlated (ESM Figs. S7 and S8). Identified discrepancies may be due to the inherent insensitivity of NMR and the difficulty of accurately fitting the compounds from within a complex mixture where not all the contributions have been identified. This supposition is generally confirmed by looking at the HPLC and NMR measurement correlation for each GbE constituent on a compound-by-compound basis in ESM Fig. S8. The correlation of the more abundant terpene trilactones (bilobalide and ginkgolide A) is better than that of the less abundant ginkgolides, B, C, and J. In general, there appears to be a slight bias to the NMR data showing a lower value than the corresponding HPLC constituent measurements. The poor correlation for rutin is strongly driven by the very high NMR values in unfinished samples, A, B, C, and F (ESM Fig. S7). However, the NMR data appears to be a very good fit with both sugar and flavonol resonances accurately matching the standard compound intensity (Fig. 5). The PCA plots in Fig. 8 did not match as closely, again emphasizing that subtle changes in the variance and range of values measured can drastically alter the score plot. The clustering of samples in the scores plots is related, but the differences in values measured account for different clustering. However, the loading plots did show similarities in the compounds that were highly correlated and could be used to differentiate characteristic versus uncharacteristic samples. This provides a nice template for how other botanicals could be compared. For example, with a few characteristic samples in the scores plot, the loading plot shows which compounds are differentiating the groups. The loading plots in Fig. 8 show some clear similarities. For example, the primary drivers that differentiate the samples are the rutin content, which is mostly orthogonal to the highly correlated bilobalide, and ginkgolide A content. The other ginkgolides are not strong drivers of differentiation. Also of note, the variance in quercetin and kaempferol typically negatively correlates with both the rutin and bilobalide/ginkgolide A groups. This can be read as ginkgolide A and bilobalide content pointing toward a sample being characteristic of GbE, and very high rutin content pointing toward a sample being uncharacteristic. This finding is unsurprising given that terpene trilactones are unique to *Ginkgo biloba* and addition of a single flavonol to mimic the 24% flavonol profile represents a known route of adulteration [3]. Taken together, targeted measurements of only rutin, ginkgolide A or bilobalide, and kaempferol or quercetin could provide excellent differentiation of characteristic versus uncharacteristic GbE using unhydrolyzed samples. The clustering in Figs. 6 and 8, and the bilobalide versus rutin content provide the basis for the annotation of characteristic versus uncharacteristic in Table 3.

Evaluation of targeted chemical analysis through the lens of HPTLC results can provide another layer of information. For example, unhydrolyzed samples in the HPLC analysis with the highest total flavonol content, ranging from 3.9 to 9.1%, corresponded to those labeled uncharacteristic of GbE in the HPTLC assay, while samples labeled consistent with GbE in the HPTLC assay had lower amounts, ranging from 0.7 to 1.1% w/w. Quercetin was the largest aglycone measured across all unhydrolyzed samples with values ranging from 3.5 to 6.4% in uncharacteristic samples, and ~ 0.5% in characteristic samples. Interestingly, neither the NTP unfinished GbE samples, characteristic unfinished GbE products, finished products, nor the NIST unfinished GbE SRM was significantly different than the uncharacteristic samples (24.2% w/w) after hydrolysis.

A major goal of this work was to determine the quality of the unfinished NTP GbE test article and better understand how widely data generated in toxicity and carcinogenicity studies with that specific GbE could be extrapolated to other GbE products. Comparison of the NTP test article with the finished and unfinished products and the NIST unfinished GbE SRM (U) using each method showed it to be most similar to other GbE samples identified as characteristic and the NIST unfinished GbE SRM (Table 3). Non-targeted chromatograms of the unhydrolyzed NTP test article were most similar to unfinished products L and T and the NIST unfinished GbE SRM (U; Fig. 1). Quantitation of individual GbE constituents in the targeted approach yielded small differences in the amounts of bilobalide and ginkgolide A, with the NTP test article being somewhat higher than other characteristic unfinished GbE products and the NIST unfinished GbE SRM (U). However, flavonol glycoside content of the hydrolyzed NTPtest article was similar to the other characteristic unfinished products, including the NIST unfinished GbE SRM (U). The HPTLC analysis of the NTP test article identified it as characteristic of GbE, along with unfinished product T and the NIST unfinished GbE SRM (U; Fig. 7). PCA of the non-targeted data grouped the NTP test article samples with unfinished products L and T, the NIST unfinished SRM (U), and finished products W and Z (Fig. 8). Taken together, these findings support the use of the NTP test article as a high-quality GbE sample representative of other characteristic GbE samples. On the other hand, the results also indicate that the data generated likely should not be applied to uncharacteristic unfinished GbE samples (e.g., A, B, C, G, and H). These findings are consistent with our previous work that incorporated a biological activity evaluation of GbE samples [2]. Although data from the HPTLC work suggested that some samples contained *Sophora japonica* and there were indicators from all methods that pure flavonol glycoside could have been added to select samples (e.g., S), we did not further identify the composition of the uncharacteristic GbE samples and cannot speculate as to whether they would result in more or less toxicity than the NTP unfinished GbE product in a chronic exposure scenario.

## Conclusion

*Ginkgo biloba* provided an excellent test case for botanical comparisons because there are a number of compounds in the extracts unique to this species and there are NIST SRMs and high-quality finished products available for comparison. Our work shows general agreement on characteristic and uncharacteristic GbE sample classification of finished and unfinished products between all of the methods used (Table 3). Therefore, a simple check for the presence, absence, or amount of these unique compounds in unhydrolyzed GbE samples could be sufficient to determine authenticity. Importantly, this survey found that the majority of unfinished GbE samples were not characteristic of high-quality GbE, indicating a problem in the supply chain. It is important to recognize that botanicals for which the constituents are largely unknown might require a more sophisticated comparison of their content using a combination of these, or other, additional methods for authenticity, or more samples to make better qualitative comparisons. In those cases, a combination of non-targeted and targeted approaches using HPLC and HPTLC and chemometric analyses are recommended. Using our approach, the NTP test article was found to be most similar to two other characteristic unfinished products (L and T), finished products containing EGb761 (X, Y, and Z), and the NIST unfinished GbE SRM.

## Electronic supplementary material

ESM 1 (PDF 1314 kb).

## References

[1] Rider CV, Walker NJ, Waidyanatha S (2018). Getting to the root of the matter: challenges and recommendations for assessing the safety of botanical dietary supplements. Clin Pharmacol Ther.

[2] Catlin NR, Collins BJ, Auerbach SS, Ferguson SS, Harnly JM, Gennings C (2018). How similar is similar enough? A sufficient similarity case study with Ginkgo biloba extract. Food Chem Toxicol.

[3] van Beek TA, Montoro P (2009). Chemical analysis and quality control of Ginkgo biloba leaves, extracts, and phytopharmaceuticals. J Chromatogr A.

[4] Kressmann S, Muller WE, Blume HH (2002). Pharmaceutical quality of different Ginkgo biloba brands. J Pharm Pharmacol.

[5] Fransen HP, Pelgrom SMGJ, Stewart-Knox B, de Kaste D, Verhagen H. Assessment of health claims, content, and safety of herbal supplements containing Ginkgo biloba. Food Nutr Res. 2010;54:5221. 10.3402/fnr.v54i0.5221. 10.3402/fnr.v54i0.5221 PMC2950792 20927202

[6] U.S. Pharmacopeia. Powdered Ginkgo Extract. USP-NF: U.S. Pharmacopeia; 2009.

[7] Booker A, Frommenwiler D, Reich E, Horsfield S, Heinrich M (2016). Adulteration and poor quality of Ginkgo biloba supplements. J Herb Med.

[8] Chen P, Ozcan M, Harnly J (2007). Chromatographic fingerprint analysis for evaluation of Ginkgo biloba products. Anal Bioanal Chem.

[9] Harnly JM, Luthria D, Chen P (2012). Detection of adulterated Ginkgo biloba supplements using chromatographic and spectral fingerprints. J AOAC Int.

[10] Ding XP, Qi J, Chang YX, Mu LL, Zhu DN, Yu BY (2009). Quality control of flavonoids in Ginkgo biloba leaves by high-performance liquid chromatography with diode array detection and on-line radical scavenging activity detection. J Chromatogr A.

[11] Braquet P (1987). The ginkgolides potent platelet-activating factor antagonists isolated from Ginkgo biloba L. chemistry pharmacology and clinical applications. Drugs Future.

[12] Heads JA, Hawthorne RL, Lynagh T, Lynch JW (2008). Structure-activity analysis of ginkgolide binding in the glycine receptor pore. J Neurochem.

[13] Kondratskaya EL, Lishko PV, Chatterjee SS, Krishtal OA (2002). BN52021, a platelet activating factor antagonist, is a selective blocker of glycine-gated chloride channel. Neurochem Int.

[14] Huang SH, Duke RK, Chebib M, Sasaki K, Wada K, Johnston GAR (2004). Ginkgolides, diterpene trilactones of Ginkgo biloba, as antagonists at recombinant alpha(1)beta(2)gamma(2L) GABA(A) receptors. Eur J Pharmacol.

[15] Koch E, Jaggy H, Chatterjee SS (2000). Evidence for immunotoxic effects of crude Ginkgo biloba L. leaf extracts using the popliteal lymph node assay in the mouse. Int J Immunopharmacol.

[16] Hecker H, Johannisson R, Koch E, Siegers CP (2002). In vitro evaluation of the cytotoxic potential of alkylphenols from Ginkgo biloba L.. Toxicology..

[17] Westendorf J, Regan J (2000). Induction of DNA strand-breaks in primary rat hepatocytes by ginkgolic acids. Pharmazie..

[18] NTP. NTP Technical Report on the Toxicology and Carcinogenesis Studies of *Ginkgo biloba* Extract (CAS No. 90045-36-6) in F344/N Rats and B6C3F1/N Mice (Gavage Studies). Research Triangle Park, NC: NIEHS/NTP; 2013.

[19] Rider CV, Nyska A, Cora MC, Kissling GE, Smith C, Travlos GS (2014). Toxicity and carcinogenicity studies of Ginkgo biloba extract in rat and mouse: liver, thyroid, and nose are targets. Toxicol Pathol.

[20] Napolitano JG, Lankin DC, Chen SN, Pauli GF (2012). Complete 1H NMR spectral analysis of ten chemical markers of Ginkgo biloba. Magn Reson Chem.

[21] Kuszak AJ, Hopp DC, Williamson JS, Betz JM, Sorkin BC (2016). Approaches by the US National Institutes of Health to support rigorous scientific research on dietary supplements and natural products. Drug Test Anal.

[22] Kellogg JJ, Paine MF, McCune JS, Oberlies NH, Cech NB (2019). Selection and characterization of botanical natural products for research studies: a NaPDI center recommended approach. Nat Prod Rep.

[23] Waidyanatha S, Pierfelice J, Cristy T, Mutlu E, Burback B, Rider CV, et al. A strategy for test article selection and phytochemical characterization of Echinacea purpurea extract for safety testing. Food Chem Toxicol. 2020;137:111125. 10.1016/j.fct.2020.111125. 10.1016/j.fct.2020.111125 PMC7079738 31931071

[24] Wallace ED, Oberlies NH, Cech NB, Kellogg JJ (2018). Detection of adulteration in Hydrastis canadensis (goldenseal) dietary supplements via untargeted mass spectrometry-based metabolomics. Food Chem Toxicol.

[25] Kellogg JJ, Graf TN, Paine MF, McCune JS, Kvalheim OM, Oberlies NH (2017). Comparison of metabolomics approaches for evaluating the variability of complex botanical preparations: green tea (Camellia sinensis) as a case study. J Nat Prod.

[26] Ma CY, Oketch-Rabah H, Kim NC, Monagas M, Bzhelyansky A, Sarma N (2018). Quality specifications for articles of botanical origin from the United States Pharmacopeia. Phytomedicine..

[27] Gawron-Gzella A, Marek P, Chanaj J, Matlawska I (2010). Comparative analysis of pharmaceuticals and dietary supplements containing extracts from the leaves of Ginkgo biloba L.. Acta Pol Pharm.

