# Supplementary material for: Comparison of phytochemical composition of Ginkgo biloba extracts using a combination of non-targeted and targeted analytical approaches
Source: Anal Bioanal Chem. 2020 Aug 31;412(25):6789–809. doi: 10.1007/s00216-020-02839-7 (PMC7496025; doi:10.1007/s00216-020-02839-7)
Supplement: Supplementary file 1 — (PDF 1314 kb). [file 216_2020_2839_MOESM1_ESM.pdf]

Analytical and Bioanalytical Chemistry

Electronic Supplementary Material

Comparison of phytochemical composition of *Ginkgo biloba* extracts using a combination of non-targeted and targeted analytical approaches

Bradley J. Collins, Season Kerns, Kristin Aillon, Geoffrey Mueller, Cynthia V. Rider,
Eugene F. DeRose, Robert E. London, James Harnly, Suramya Waidyanatha

Supplementary Tables

Table S1 Information for *Ginkgo biloba* Extract Products Procured from Various Suppliers

GbE ID	Supplier	Supplier Chemical Name	Supplier Lot	Sample Type	Supplier COA
A	1	Ginkgo biloba P.E. 24%	Gbo20140907-2	Unfinished Product	Flavonols 24%
B	1	Ginkgo biloba P.E. 10:1	Gbo20140907-4	Unfinished Product	Extract Ratio
C	1	Ginkgo biloba P.E. 4:1	Gbo20140907-3	Unfinished Product	Extract Ratio
D	2	Ginkgo biloba P.E. 24/6	CMYX-A-01272	Unfinished Product	Flavonols 24%, Terpenes 6%
E	3	Ginkgo biloba Leaves PE 24/6	B408017	Unfinished Product	Flavonols 24%, Terpenes 6%
F	4	Ginkgo biloba P.E. 24%	Gbo2014032	Unfinished Product	Flavonols 24%
G	5	Ginkgo biloba Leaf Powder	CYX-A-00590	Unfinished Product	None
H	5	Ginkgo biloba Leaf Extract 4:1	CYX20120201	Unfinished Product	Extract Ratio
I	5	Ginkgo biloba P.E. 24/6	GB2014818	Unfinished Product	Flavonols 24%, Terpenes 6%
J	6	PureLeaf™ Ginkgo biloba Extract	EN-140426	Unfinished Product	None
K	7	Ginkgo biloba Dry Extract	478131	Unfinished Product	None
L	8	Ginkgo biloba EPG 246	60814002	Unfinished Product	Flavonols 24%, Terpenes 6%
M	9	Ginkgo biloba PE 4:1	C276/086/A14	Unfinished Product	Extract Ratio
N	9	Ginkgo Leaf PE 24% Flavone	D072/030/A14	Unfinished Product	Flavonols 24%, Terpenes 6%

		Glycosides / 6% Ginkgolides			
O	10	Ginkgo biloba Extract 24/6	1212134	Unfinished Product	Flavonols 24%, Terpenes 6%
P	11	Ginkgo biloba Powder Extract	O-140303	Unfinished Product	None
Q	12	Ginkgo Extract	RMGK-090614	Unfinished Product	None
R	13	Ginkgo biloba Extract 24/6	GIN24D-140610	Unfinished Product	Flavonols 24%, Terpenes 6%
S	14	Ginkgo biloba Extract USP	20131205	Unfinished Product	?
T	15	Ginkgo 24/6/5	14090785	Unfinished Product	Flavonols 24%, Terpenes 6%
U	NIST	Ginkgo biloba (Extract)	SRM 3247	SRM	Flavonols 24%, Terpenes 6%, Ginkgolic Acids <1%
V	NIST	Ginkgo-Containing Tablets	SRM 3248	SRM	Flavonols 24%, Terpenes 6%, Ginkgolic Acids <1%
W	16	Ginkgo Standardized (60 mg GbE per capsule; 340 mg Gotu Kola per capsule)	20009556	Finished Product	Flavonols 24% Terpenes 6% Ginkgolic Acids <1%
X	16	Ginkgold tablets (60 mg GbE per tablet)	20031047	Finished Product	Flavonols 24%, Terpenes 6%, Ginkgolic Acids <1%
Y	16	Ginkgold MAX tablets (120 mg GbE per tablet)	20024869	Finished Product	Flavonols 24%, Terpenes 6%, Ginkgolic Acids <1%
Z	16	Ginkgold MAX tablets (120 mg GbE per tablet)	20031396	Finished Product	Flavonols 24%, Terpenes 6%, Ginkgolic Acids <1%

Table S2 Summary of Linear Regression Parameters for HPLC Component Standard Curves

Analyte	Correlation Coefficient (r)	Effective Curve Range[1] (~ µg/mL)	Nominal LOD[2] (Wt%; g/g)	Nominal LOQ[3] (Wt%; g/g)
Bilobalide	≥ 0.99	11.4 – 64.6	0.32	1.44
Ginkgolide A	≥ 0.99	10.6 – 54.8	0.21	1.33
Ginkgolide B	≥ 0.99	10.3 – 56.8	0.19	1.28
Ginkgolide C	≥ 0.99	10.7 – 55.2	0.13	1.34
Ginkgolide J	≥ 0.99	12.6 – 53.6	0.30	1.58
Rutin	≥ 0.999	3.82 – 72.3	0.003	0.48
Quercetin	≥ 0.999	4.31 – 76.0	0.004	0.54
Kaempferol	≥ 0.999	4.54 – 80.6	0.01	0.72
Isorhamnetin	≥ 0.99	2.32 – 35.5	0.01	0.35
Ginkgolic Acid C15	≥ 0.99	1 - 500	0.00002	0.00007
Ginkgolic Acid C17	≥ 0.99	1 - 500	0.00001	0.00019
Ginkgotoxin	≥ 0.9999	10 – 2,000	0.00006	0.00659

Criteria: $r \geq 0.99$; %RE ≤ 25%

[1] The Effective Curve Range was determined by calculating the mean across all analysis days.

[2] Nominal LOD = 3 x signal to noise ratio of the blank chromatogram at or near the retention time of the GbE constituent, expressed as Wt%; g/g.

[3] Nominal LOQ = The nominal value of the lowest concentration standard expressed as Wt%; g/g.

Table S3 Percent by Weight of Hydrolyzed GbE Constituents Quantitated by HPLC

			Terpene Trilactones					Flavonols[1]		
Source/Type[1]	GbE ID	Bilobalide	A	B	C	J	Rutin	Quercetin	Kaempferol	Isorhamnetin
NTP UFP	1	7.95	4.22	1.62	1.89	**1.03**	0.71	6.00	4.19	0.59
NTP UFP	1A	7.86	3.94	1.57	1.97	**1.09**	0.85	5.80	4.04	0.53
NTP UFP	1A[3]	8.73	4.90	2.19	1.80	2.42	ND	6.56	3.86	1.06
NTP UFP	1F	7.95	4.26	1.66	2.07	**1.13**	0.78	5.84	4.07	0.53
1 UFP	A	**1.28**	ND	ND	ND	1.77	BLOD	1.62	**0.09**	**0.17**
1 UFP	B	ND	ND	ND	ND	ND	**0.05**	1.21	**0.08**	**0.15**
1 UFP	C	ND	ND	ND	ND	ND	**0.01**	0.94	**0.07**	**0.14**
2 UFP	D	**1.28**	2.60	1.60	2.79	**1.18**	1.11	3.77	3.35	**0.20**
3 UFP	E	1.50	2.13	1.46	2.60	**1.21**	1.21	4.08	2.37	**0.22**
4 UFP	F	ND	ND	ND	ND	ND	**0.04**	1.15	**0.07**	**0.14**
5 UFP	G	**0.55**	**0.68**	**0.85**	ND	**0.82**	ND	ND	ND	ND
5 UFP	H	ND	ND	ND	ND	ND	ND	ND	ND	ND
5 UFP	I	1.53	2.45	1.50	1.86	**1.08**	0.69	5.42	2.15	**0.27**
6 UFP	J	2.26	3.78	1.97	1.62	**1.08**	**0.46**	5.23	3.86	**0.30**
7 UFP	K	3.33	2.44	1.40	1.87	**1.10**	1.37	4.13	3.08	**0.28**
8 UFP	L	3.35	2.72	1.37	1.66	**1.07**	3.20	1.54	1.01	**0.25**

9 UFP	M	**1.22**	ND	ND	ND	ND	ND	0.55	**0.45**	**0.16**
9 UFP	N	1.89	4.09	1.90	1.38	**0.95**	0.59	4.69	2.67	**0.34**
10 UFP	O	2.77	2.82	1.69	1.51	**1.08**	**0.20**	7.52	3.00	0.79
11 UFP	P	ND	**0.92**	**0.78**	**0.85**	ND	ND	2.90	8.43	0.37
12 UFP	Q	1.91	2.38	1.78	2.83	**1.22**	**0.16**	4.79	5.32	0.63
13 UFP	R	2.14	2.38	1.80	1.78	**1.00**	ND	4.09	5.31	0.51
14 UFP	S	2.73	1.83	**0.96**	1.61	**1.03**	**0.40**	5.85	5.33	0.66
15 UFP	T	2.45	2.13	1.46	1.39	**0.95**	0.64	4.37	4.82	1.82
NIST SRM	U	2.75	1.71	**1.07**	1.77	**1.08**	1.10	4.79	4.35	0.75
NIST SRM	U[3]	4.21	2.28	1.60	1.75	2.29	ND	4.74	3.26	0.95
NIST SRM	V	**0.91**	**0.89**	**0.72**	**0.84**	**0.83**	**0.21**	0.91	0.99	**0.26**
16 FP	W	5.55	2.96	3.50	1.74	**1.23**	2.25	4.71	4.43	0.40
16 FP	X	4.38	2.60	1.64	2.03	**1.15**	2.25	4.18	3.60	0.69
16 FP	Y	4.60	2.71	1.45	2.11	**1.31**	2.14	4.31	3.66	0.62
16 FP	Z	4.46	2.45	1.37	1.85	**1.26**	1.92	4.64	4.01	0.63

[1]Values reported for quercetin, kaempferol and isorhamnetin are aglycone concentrations. Glycoside values may be obtained by multiplying the quercetin, kaempferol and isorhamnetin values by 2.504, 2.588, and 2.437, respectively. Values listed in bold are below LOQ. See Table S2 for LOQ for each GbE constituent. ND: Not detected; BLOD: Below the limit of detection. See Table S2 for LOD for each GbE constituent.

[2]UPF = Unfinished Product; FP = Finished Product; SRM = Standard Reference Material

[3]Repeat Analysis on a different instrument and on a different day

Table S4 Weight Percent of Ginkgolic Acids and Ginkgotoxin in Unhydrolyzed and Hydrolyzed GbE Samples

		Unhydrolyzed[1]			Hydrolyzed[1]		
Source/Type[2]	GbE	Ginkgolic Acid C15	Ginkgolic Acid C17	Ginkgotoxin	Ginkgolic Acid C15	Ginkgolic Acid C17	Ginkgotoxin
NTP UFP	1	0.00182	0.00021	0.00754	0.00169	**0.00018**	0.00997
NTP UFP	1A	0.00171	**0.00015**	0.00788	0.00159	**0.00015**	0.00883
NTP UFP	1A[3]	0.00128	0.00066	0.01008	0.00149	0.00052	0.01102
NTP UFP	1F	0.00160	**0.00013**	0.00799	0.00154	**0.00012**	0.00952
1 UFP	A	0.00030	0.00059	**0.00304**	0.00027	0.00045	**0.00388**
1 UFP	B	0.00041	0.00069	**0.00302**	0.00049	0.00075	**0.00394**
1 UFP	C	0.00034	0.00062	**0.00306**	0.00076	0.00102	**0.00391**
2 UFP	D	0.00091	0.00089	0.01022	0.00059	0.00061	0.01000
3 UFP	E	0.00010	0.00022	**0.00265**	**0.00006**	0.00019	**0.00266**
4 UFP	F	0.00047	0.00076	**0.00283**	0.00108	0.00144	**0.00366**
5 UFP	G	0.12690	0.21393	**0.00393**	0.13550	0.22045	**0.00417**
5 UFP	H	0.00037	0.00053	0.00909	0.00027	0.00040	0.01004
5 UFP	I	0.00208	0.00266	0.01625	0.00161	0.00194	0.01694
6 UFP	J	0.00036	0.00048	0.06146	0.00039	0.00047	0.05976
7 UFP	K	0.00031	0.00027	0.08466	0.00019	**0.00013**	0.08407
8 UFP	L	BLOD	BLOD	0.04466	BLOD	BLOD	0.04446
9 UFP	M	0.00034	0.00045	0.01045	0.00024	0.00030	0.01156
9 UFP	N	0.08338	0.05940	0.04383	0.08201	0.05677	0.04341
10 UFP	O	0.00048	0.00043	0.05262	0.00075	**0.00011**	0.08991
11 UFP	P	0.00045	**0.00011**	0.01409	0.00045	**0.00010**	0.01468
12 UFP	Q	0.00115	0.00077	0.05065	0.00078	0.00042	0.06155
13 UFP	R	0.00030	**0.00012**	0.03211	0.00025	**0.00012**	0.03850
14 UFP	S	BLOD	BLOD	0.06728	BLOD	BLOD	0.11083

15 UFP	T	**0.00002**	**0.00001**	0.04728	BLOD	BLOD	0.05039
NIST SRM	U	0.00030	0.00024	0.05585	0.00012	**0.00010**	0.05304
NIST SRM	U[3]	0.00028	0.00026	0.03779	0.00019	**0.00015**	0.03879
NIST SRM	V	BLOD	BLOD	0.01047	BLOD	BLOD	**0.01032**
16 FP	W	**0.00002**	**0.00003**	0.04542	BLOD	BLOD	0.04291
16 FP	X	BLOD	BLOD	**0.00483**	BLOD	BLOD	**0.00030**
16 FP	Y	BLOD	BLOD	**0.00392**	BLOD	BLOD	BLOD
16 FP	Z	BLOD	BLOD	**0.00425**	BLOD	BLOD	BLOD

[1]Values listed in bold are below LOQ but above the limit of detection. See Table S2 for LOQ for each GbE constituent.

BLOD: Below the limit of detection. See Table S2 for the LOD for each GbE. constituent

[2]UFP = Unfinished Product; FP = Finished Product; SRM = Standard Reference Material
[3]Repeat Analysis on a different instrument and on a different day

Table S5 Comparison of Found vs. Reported GbE Constituents Concentrations in Hydrolyzed NIST SRM

	**Bilobalide**	**Ginkgolides (mg/g)**				**Flavonols (mg/g)**		
	**(mg/g)**	**A**	**B**	**C**	**J**	**Quercetin**	**Kaempferol**	**Isorhamnetin**
NIST 3247 Reported Values	28.5	11.6	5.92	12.4	3.9	45.1	40.8	10.8
Sample U	27.5	17.1	10.7	17.7	10.8	47.9	43.5	7.5
Sample U[1]	42.1	22.8	16.0	17.5	22.9	47.4	32.6	9.5

[1] Duplicate sample run on a different instrument and on a different day.

Supplementary Figures

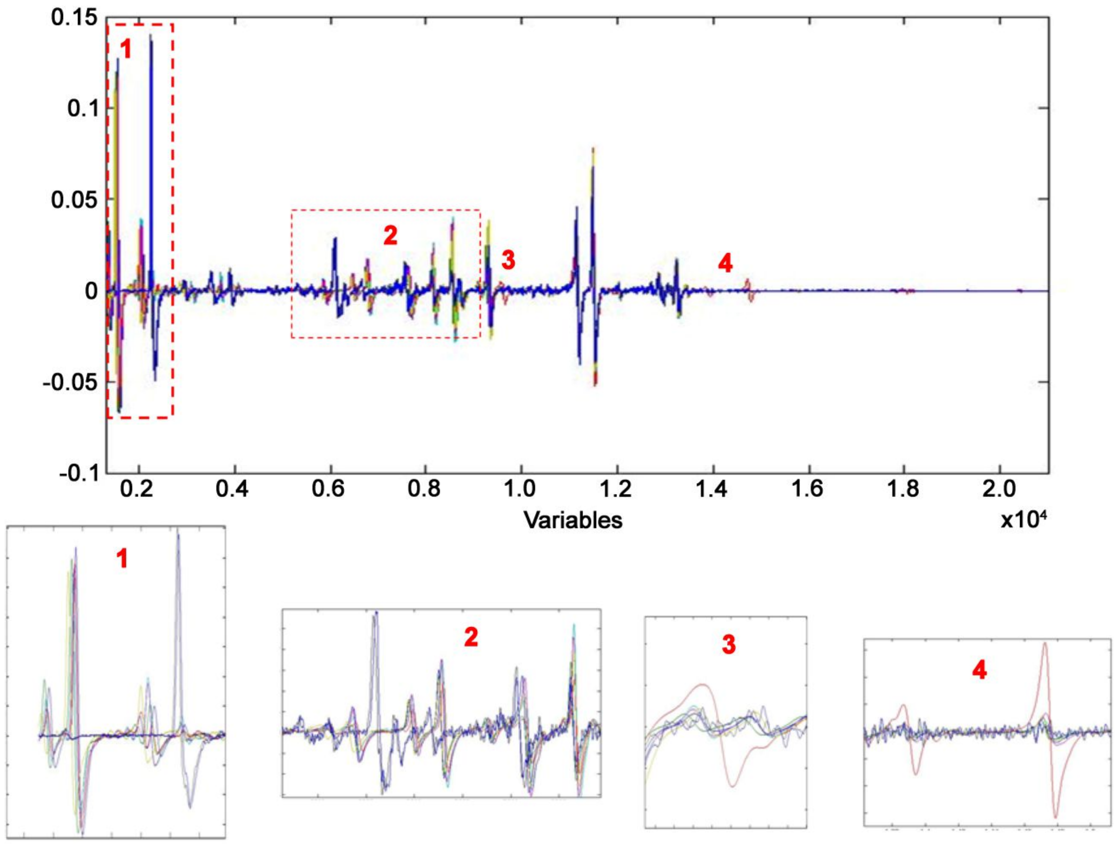

Fig. S1 Chromatograms for the 8 samples in cluster A (samples U, V, W, X ,Y, Z, 1, and 1F)

Insets 1 and 2 highlight the differences between the NIH archived samples and the rest of the samples

Insets 3 and 4 highlight the difference between sample W and the rest of the samples.

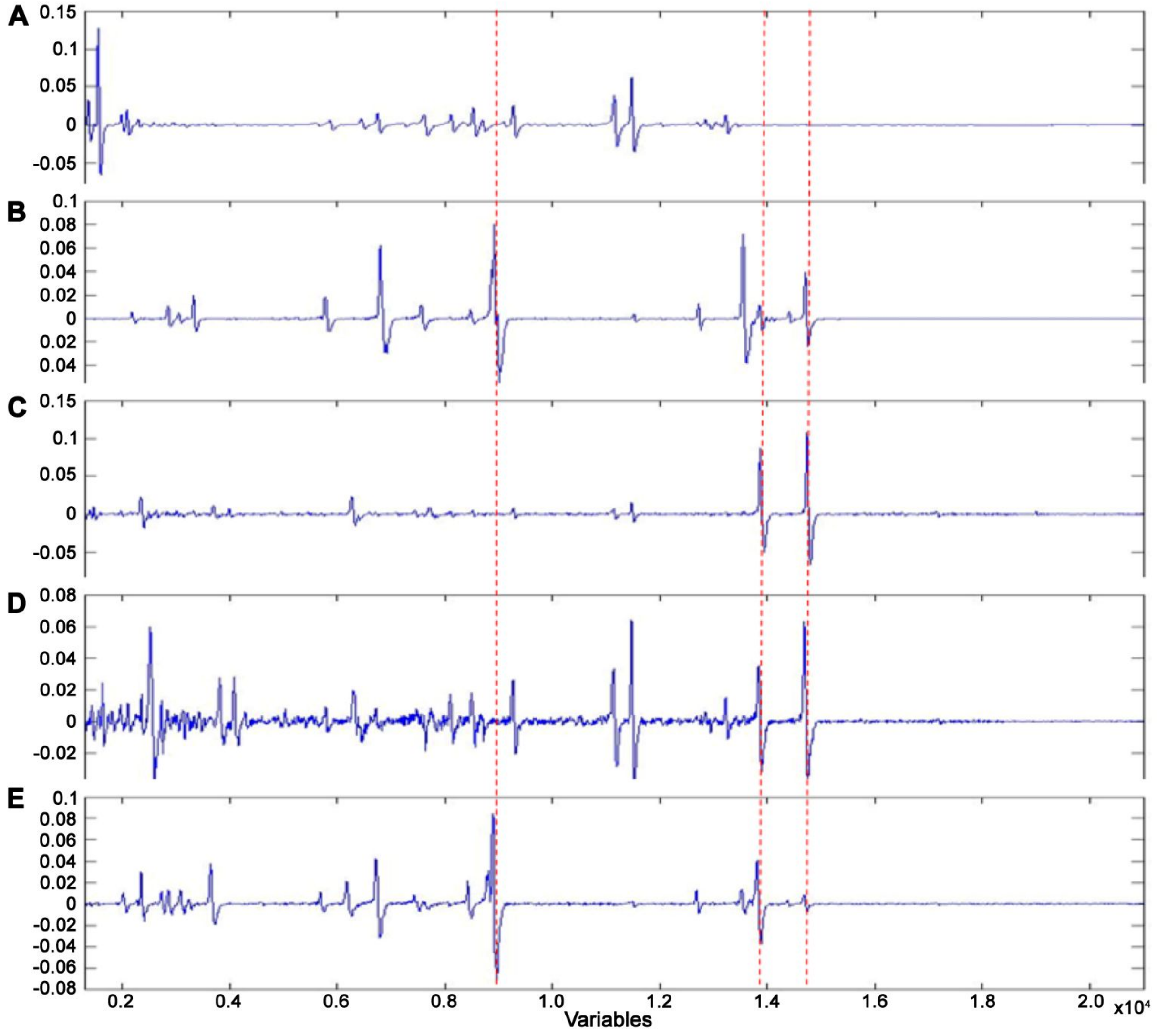

Fig. S2 Chromatograms for a sample from each cluster in Figure 1A: (A) sample U from cluster A, (B) sample P from cluster B, (C) sample J from cluster C, (D) sample K from cluster D, and (E) sample D from cluster E

Differences in the sample composition are emphasized by the vertical dashed lines

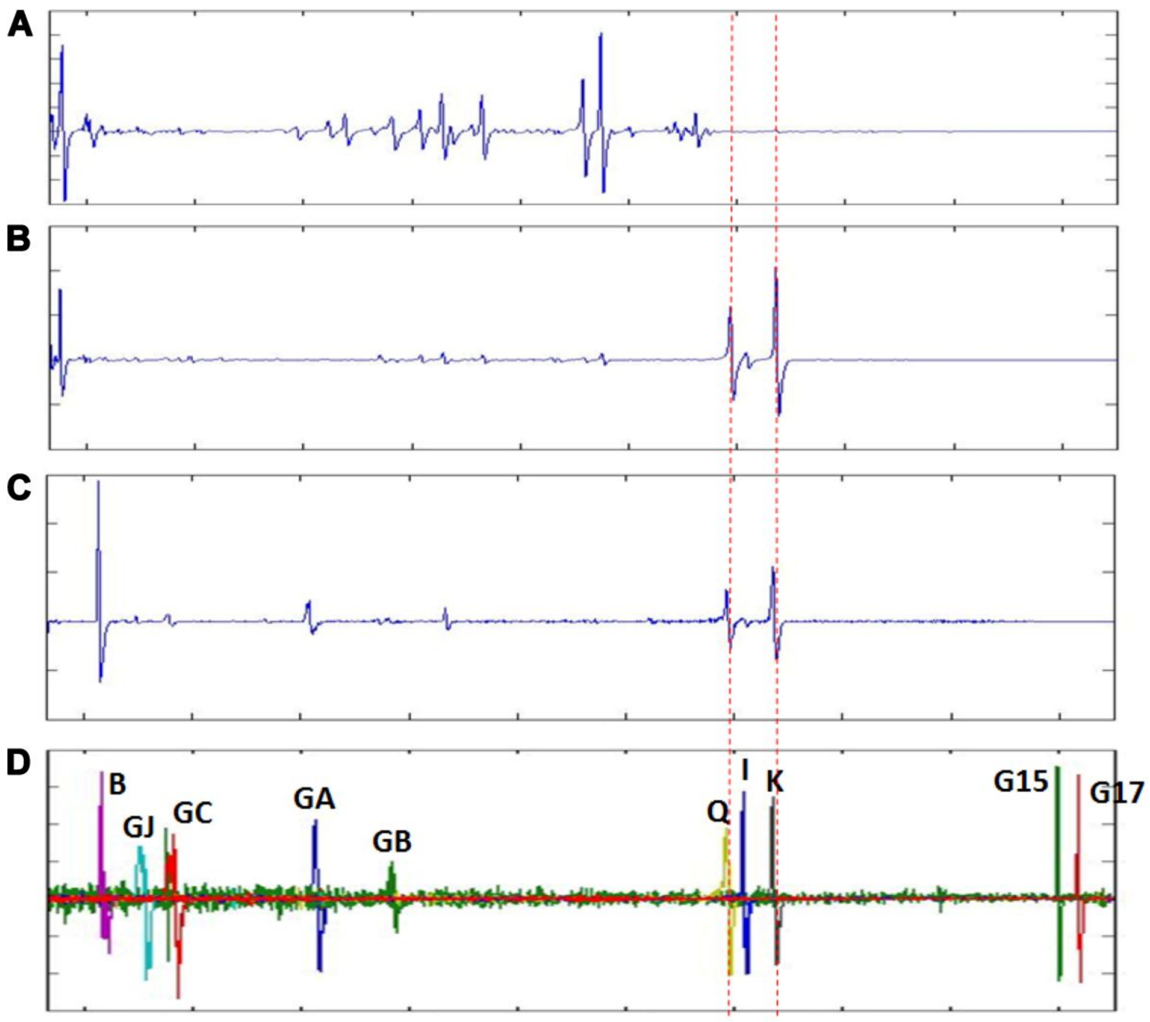

Fig. S3 Chromatograms illustrating the difference in sample composition induced by hydrolysis: (A) averaged, un-hydrolyzed chromatogram for samples U, V, X, Y, and Z, (B) averaged, hydrolyzed chromatogram for samples U, V, X, Y, and Z, (C) averaged, hydrolyzed chromatogram for samples 1 and 1F, and (D) combined chromatograms for reference standards Notations for the standards are B=bilobalide, GA=ginkgolide A, GB=ginkgolide B, GC=ginkgolide C, GJ=ginkgolide J, Q=quercetin, I=isorhamnetin, K=kaempferol, G15=ginkgolic acid 15, and G17=ginkgolic acid 17. The vertical dashed lines dente the retention times for quercetin and kaempferol.

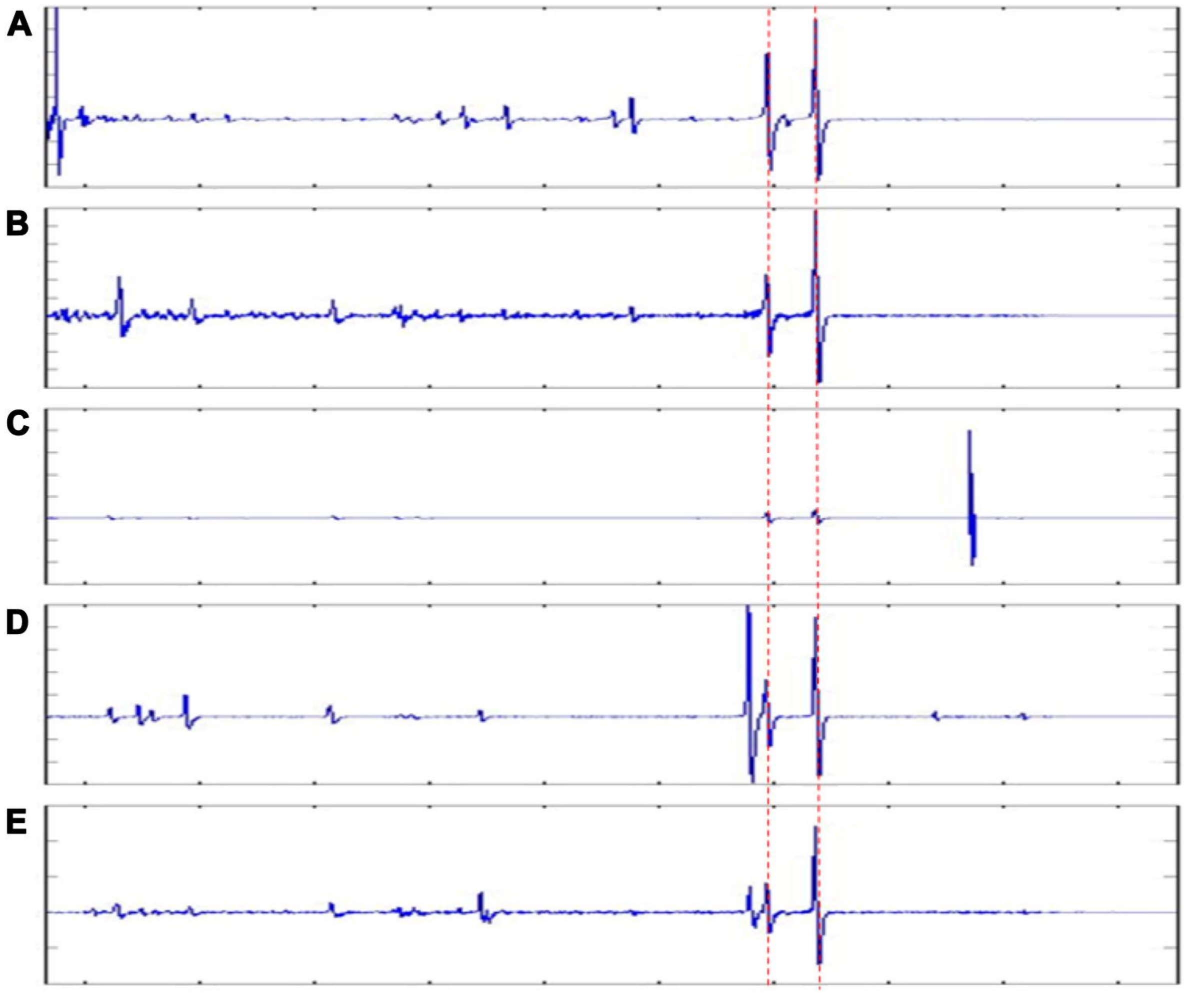

Fig. S4 Chromatograms for a sample from each group in Figure 1B: (A) sample U, (B) sample K, (C) sample J, (D) sample D, and (E) sample R

The vertical dashed lines denote the retention times for quercetin and kaempferol

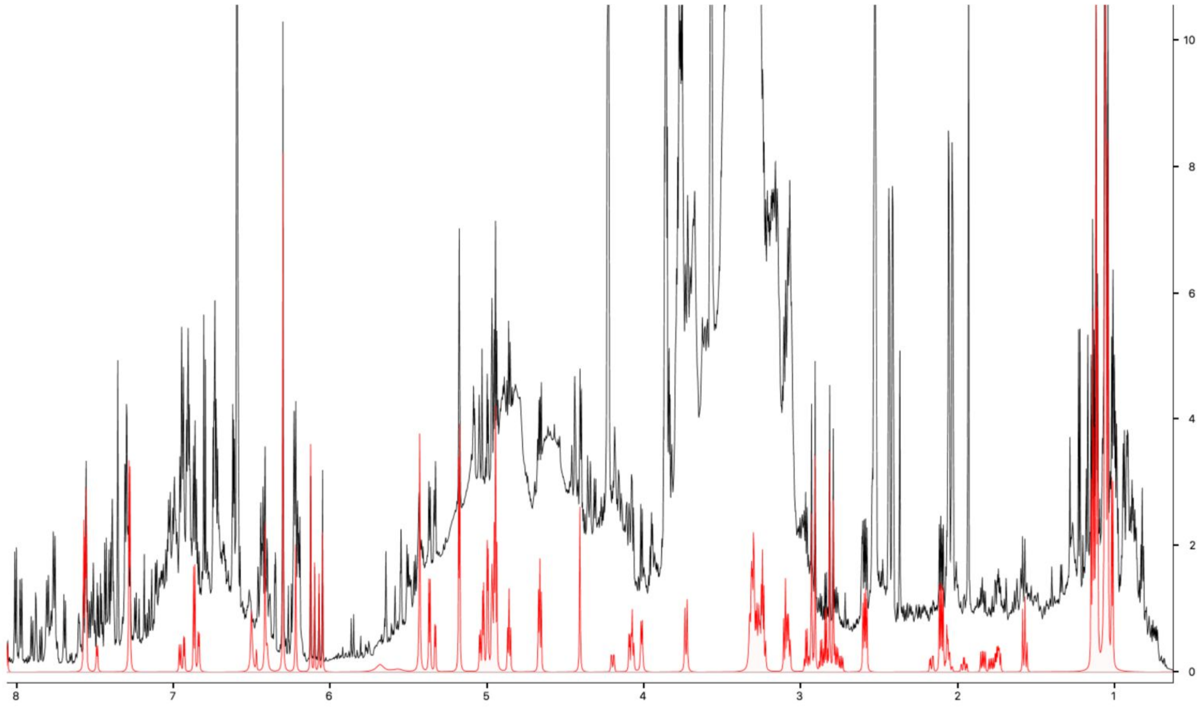

Fig. S5 Example NMR analysis of GbE-U

Black lines indicate the NMR spectra, and red lines indicate standard compounds fit in the data

X-axis is ^{1}H ppm, and the y-axis is relative intensity

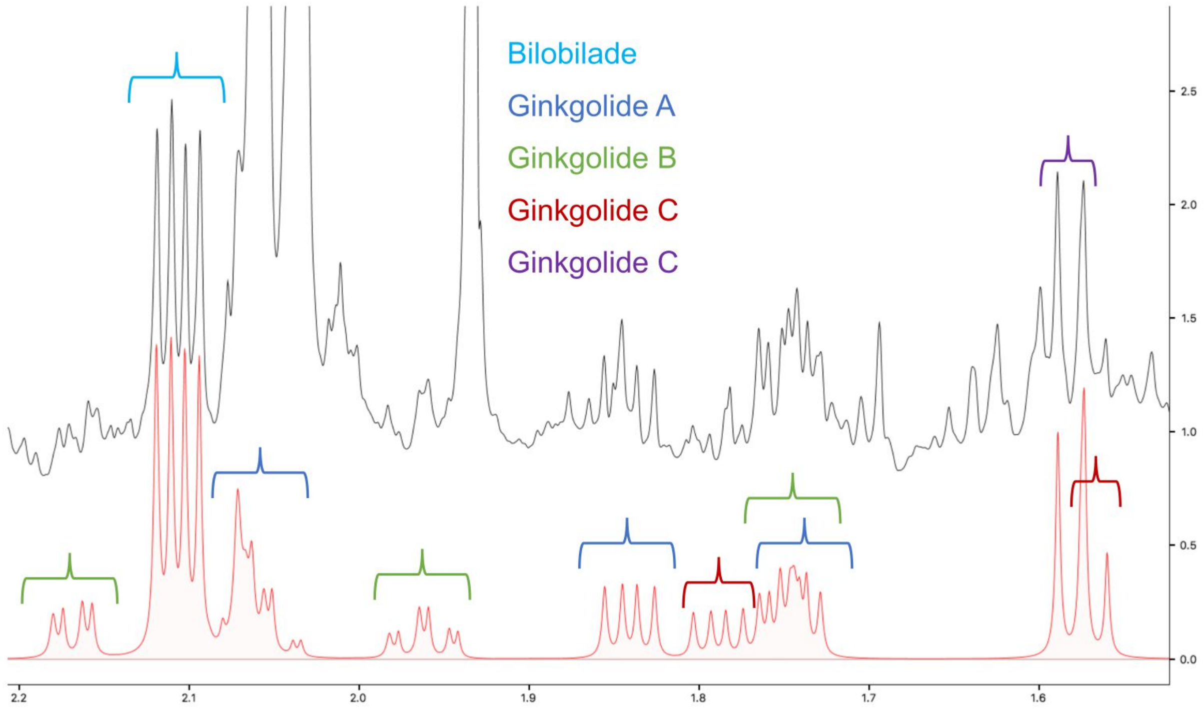

Fig. S6 Example NMR analysis of GbE-U

Black lines indicate the NMR spectra, and red lines indicate standard compounds fit in the data

Colored braces indicate peaks from bilobalide (light blue), ginkgolide A (dark blue), ginkgolide B (green), ginkgolide C (maroon), ginkgolide J (purple)

X-axis is ^{1}H ppm, and the y-axis is relative intensity.

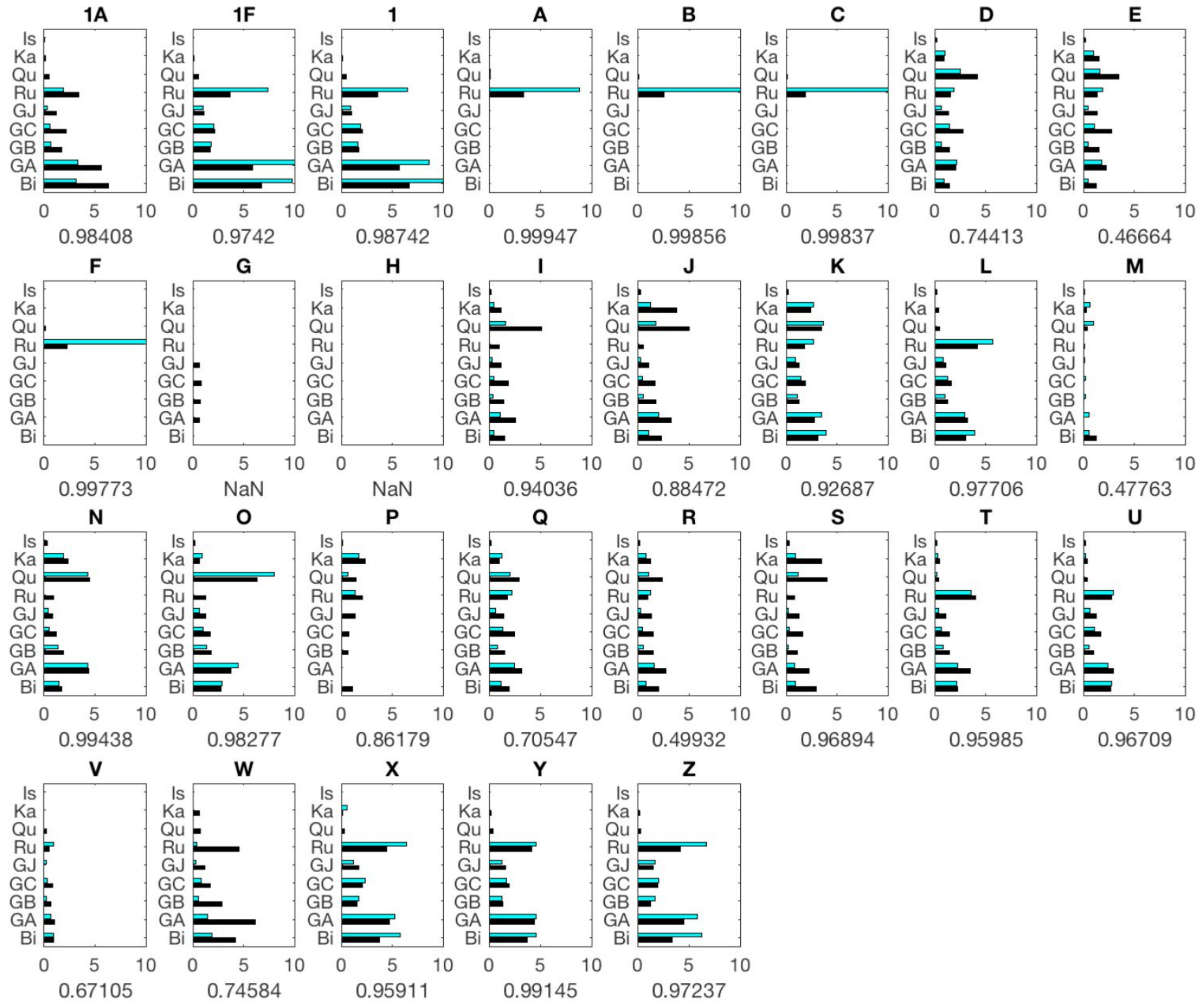

Fig. S7 Direct comparisons of the LC and NMR results of weight percent

The 29 GbE samples are labeled at the top

The x-axis is weight percent of the compound of the total sample (g/g)

The y-axis shows the compound abbreviations: Is (Isorhamnetin), Ka (kaempferol), Qu (quercetin), Ru (rutin), GJ (ginkgolide J), GC (ginkgolide C), GB (ginkgolide B), GA (ginkgolide A), Bi (Bilobilade)

Cyan bars are the NMR measurement and black bars are the LC measurement

Below each graph is the correlation coefficient for all but Is, unless other values were zero where NaN is indicated.

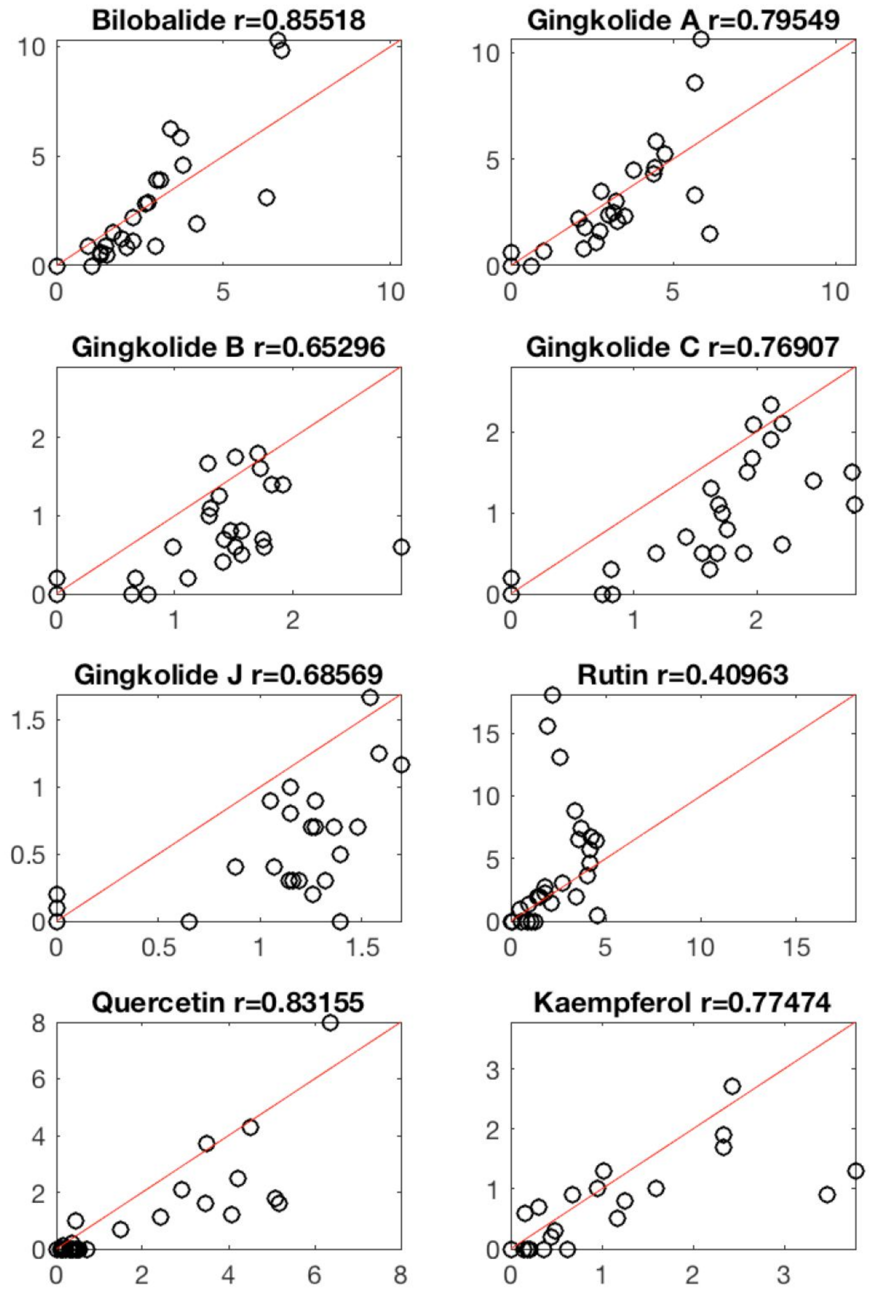

Fig. S8 Comparison of the weight percent (ppm) measure by LS and NMR

The LC (x-axis) and NMR (y-axis) weight percent in ppm are compared for the 29 samples measured

The graphs are titled with the compound and correlation coefficient of the measurements
